# VHL-recruiting PROTAC attenuates AKI-CKD transition via simultaneous degradation of Smad3 and stabilization of HIF-2α

**DOI:** 10.1038/s41419-026-08726-w

**Published:** 2026-04-10

**Authors:** Yuyi Ruan, Dan Wang, Yuzhu Xu, Jiayi Yang, Yutong Chen, Jinjin Fan, Sydney C. W. Tang, Wei Chen, Xin Wang

**Affiliations:** 1 Department of Nephrology, The First Affiliated Hospital, Sun Yat-sen University, Guangzhou, China; 2 NHC Key Laboratory of Clinical Nephrology (Sun Yat-Sen University) and Guangdong Provincial Key Laboratory of Nephrology, Guangzhou, China; 3 Division of Nephrology, Department of Medicine, School of Clinical Medicine, The University of Hong Kong, Queen Mary Hospital, Hong Kong, China

**Keywords:** Acute kidney injury, Chronic kidney disease

## Abstract

Acute kidney injury (AKI) impairs renal function in the short term and may eventually progress to chronic kidney disease (CKD) in the long term. The activation of Smad3 and an imbalance in hypoxia-inducible factors-α (HIF-α) expression constitute vital mechanisms leading to the AKI-CKD transition. We have designed a Smad3-targeted Proteolysis-Targeting Chimera (PROTAC) named P1705434, which recruited VHL to degrade Smad3 and meanwhile stable HIF-2α levels. We established a cisplatin nephrotoxicity model and folic acid nephropathy (FAN) model to explore its role and possible mechanisms in the early stage and development of AKI. The results demonstrated that P1705434 alleviated inflammation and fibrosis in progressing AKI by degrading Smad3 and increasing HIF-2α. This was confirmed in both the cisplatin nephrotoxicity and FAN mice models, as evidenced by the reduction percentage of maladaptive proximal tubular cells (PT) and down-regulation of the TNF pathway, which ameliorated injury in S3-PT. Furthermore, we identified a transitional collecting duct (tCD) cell type that had a trend to differentiate into fibroblast but P1705434 treatment reduces the propensity of tCD cells and mitochondrial injury in CD cells by up-regulating the oxidative phosphorylation (OXPHOS) pathway.

## Introduction

Acute kidney injury (AKI) can be caused by drugs, toxins, and infections [1]. AKI affects renal function in the short term and may be life-threatening in severe cases, and in the long term, can lead to a sustained decline in renal function in the absence of early intervention, progressing to chronic kidney disease (CKD), leading to chronic renal failure. AKI-CKD transition is the eventual progression of AKI. Maladaptive tubular repair, persistent vascular injury, and inflammation-induced fibrosis after kidney injury may be the underlying causes of the progression of AKI-CKD transition [2]. Currently, AKI lacks specific pharmacological prevention and treatment.

The TGF-β1/Smad pathway plays an important role in the maladaptive repair of AKI and the progression to CKD. Smad3 is a central protein in the TGF-β1 pathway, and activated Smad3 promotes collagen deposition and myofibroblast activation to cause maladaptive repair of AKI, and induces the progression from AKI to CKD, as well as renal fibrosis [3]. Blocking the TGF-β1/Smad3 pathway has been shown to be effective in alleviating AKI-CKD transition by various methods [4]. Current research on TGF-β1/Smad3 has focused on the role of the TGF-β pathway in CKD and renal fibrosis rather than AKI.

When AKI occurs, renal cells suffer from hypoxia, and hypoxia-inducible factors-α (HIF-α) play a role in regulating the organism in homeostatic balance [5]. HIF-α are transcription factors, and the protein levels can be rapidly regulated by the ubiquitin-proteasome system (UPS). Von Hippel-Lindau (VHL) is the predominant E3 ligase of HIF-α in its UPS pathway. Two isoforms of HIF-α are current studies focusing on: HIF-1α and HIF-2α. Due to differences in downstream target genes, imbalance in HIF-1α and HIF-2α expression is an important mechanism leading to the progression of AKI. HIF-1α increases in the early phase of hypoxia to promote renal fibrosis, while HIF-2α increases in the late phase of hypoxia to inhibit renal fibrosis and inflammation [6]. Activation of HIF-1α during acute renal ischemia or hypoxia may attenuate renal injury by promoting angiogenesis and tissue repair, and upregulation of HIF-2α may exert a similar nephroprotective effect during acute ischemia [7]. However, sustained HIF-1α activation can have deleterious effects on the kidneys, which are mediated through the renal tubular epithelial-mesenchymal transition (EMT), promoting renal fibrosis and renal inflammation through the upregulation of pro-inflammatory cytokines as well as the conversion of macrophages from M1 to M2 subtypes. By contrast, activation of HIF-2α is nephroprotective through the inhibition of inflammatory responses and macrophage polarization [8]. Therefore, amelioration of the imbalance between HIF-1α and HIF-2α expression is a key approach to protect the kidney, alleviate AKI, and inhibit renal fibrosis.

Proteolysis-Targeting Chimeras (PROTAC), as a novel form of small molecule drugs, are composed of a protein of interest (POI)-recognizing ligand, a linker, and an E3 ligase-recognizing ligand [9]. PROTAC recruits specific E3 ligases through ligands to ubiquitinate the target proteins, which are then degraded through the proteasome pathway [10]. Since different pathogenic proteins can be degraded through the recruitment of E3 ligases by different ligands, PROTAC has great potential for precision drug discovery and development. Currently, PROTAC technology is mainly used in the development of anti-tumor drugs, and there are no studies of PROTAC applied to AKI [11].

We previously validated the renal protective effects of Smad3-targeted PROTAC named P1705434 in CKD, which recruited VHL to degrade Smad3 and meanwhile stabilized HIF-2α level [12]. In the present study, we investigate its unexplored potential in acute kidney injury. Since the effects of P1705434 were determined by prophylactic administration, we speculated that P1705434 could be renal protective in the early stages of AKI-CKD transition, and therefore, we established the folic acid nephropathy (FAN) model and the cisplatin nephrotoxicity model to explore its role and possible prevention mechanisms on AKI development.

## Methods

### Mice and animal housing

Eight-week-old male C57BL/6 mice were obtained from GemPharmatech Co., Ltd. The animals were maintained in a specific pathogen-free (SPF) facility under a controlled 12-hour light-dark cycle. The temperature ranged from 24 to 26 °C, and the humidity ranged from 50 to 70%. Mice are raised in cages with greater than two and fewer than five mice per cage. All animal experiments were conducted in compliance with ethical guidelines and approved by the Institutional Animal Care and Use Committee (IACUC) of Sun Yat-Sen University (Approval No. SYSU-IACUC-2021-000884).

### Animal models and treatments

At 8-10 weeks of age, mice were randomly allocated into control and experimental groups (*n* = 10 per group) using a computer-generated randomization sequence (Random.org).

The experimental mice were administered a single intraperitoneal injection (i.p.) of folic acid (FA, 250 mg/kg body weight dissolved in sodium bicarbonate) for induction of AKI-CKD transition. Control group mice received an equivalent volume of sodium bicarbonate buffer as a vehicle control. To capture key phases of AKI-CKD transition, cohorts of mice were sacrificed at multiple time points after FA injection: day 2 to assess the AKI, day 14 to assess the early transition phase with the initiation of interstitial fibrosis, and day 28 to assess established chronic kidney disease with significant fibrosis. To focus on early-stage injury of AKI, the experimental group mice were injected with a single dose of cisplatin (10 mg/kg body weight in saline, i.p.) and sacrificed at day 3. P1705434 (Ontores, Zhejiang, China) was given s.c. Every 2 days in a dosage of 24 mg/kg. To investigate the effects of a combination of SIS3 and FG-4592 on FAN mice, the mice were treated with 5 mg/kg SIS3 and 10 mg/kg FG-4592 dissolved per day.

Group allocation was concealed from the investigators during the conduct of the experiment, outcome assessment, and data analysis. To minimize potential confounders, the order of treatments and measurements was randomized. The sample size of 10 mice per group was determined based on power analysis using preliminary data, ensuring a statistical power of 80% and a significance level of 0.05 to detect biologically relevant differences. The outcome measure was renal fibrosis detection and renal function.

### Blood analysis

Cystatin C levels (ab201280, Abcam, UK) were quantified using a commercially available ELISA kit following the manufacturer’s instructions. Serum biochemical data of creatinine and BUN were analyzed through an automatic method and equipment (Hitachi 717 Chemistry Analyzer, Tokyo, Japan).

### Cell lines and cell culture

Human renal proximal tubule cells (HK2, ATCC, RRID:CVCL_0302), mCCDcl1 (CTCC-001-0852, RRID:CVCL_B7HR) were cultured at 37 °C in 5% CO2 in mCCDcl1 medium (CTCC-001-085-CM, MeisenCTCC). Cells were grown to approximately 70–80% confluence and stimulated with cisplatin or P1705434 in serum-free medium. For wash-out experiments, cells were stimulated with human recombinant TGF-β1 (10 ng/mL) for 24 h. Following TGF-β1 stimulation, cells were treated with either the P1705434 or a combination of Smad3 inhibitor SIS3 (5 μM) and HIF-PHD inhibitor FG-4592 (10 μg/ml). Vehicle control groups received an equivalent volume of 0.1% DMSO. After the 24-hour treatment period, the drug-containing medium was completely removed. Cells were gently washed with phosphate-buffered saline (PBS) and replaced with fresh medium. Cells were then cultured for an additional 24 or 48 h.

### Molecular Docking

Protein data file was accessed from PBD data bank. The VHL ligand moiety of P1705434 was docked into the VHL binding pocket, and the Smad3 ligand moiety was docked into the Smad3 binding pocket. The two resulting binary complexes (VHL-P1705434 and P1705434-Smad3) were then structurally aligned based on the P1705434 scaffold using PyMOL to generate ternary complex model. The final model and protein-ligand interactions were analyzed and visualized using both Discovery Studio and PyMOL.

### Cell suspension preparation from mouse tissue

For cell isolation, fresh mouse kidney tissues were minced and enzymatically digested in DMEM supplemented with collagenase IV (1 mg/mL, Gibco), DNase I (20 U/mL, Sigma-Aldrich), and hyaluronidase (0.01%, Solarbio) for 30 min at 37 °C. After digestion, the cells were filtered through a 70 μm and 40 μm strainer. Cell pellets were then treated with RBC lysis (C3702, Beyotime) and resuspended in FACS staining buffer (PBS containing 2% FBS) for further antibody staining.

### Flow cytometry

Cell suspensions were stained with fluorescently conjugated antibodies for 30 min at 4 °C. Cells were incubated with antibodies: Alexa Fluor® 700 anti-CD45 (147716, Biolegend,USA); Brilliant Violet 510™ anti-CD11b (101245, Biolegend, USA); Brilliant Violet 421 anti-F4/80 (123131, Biolegend, USA). Cells were then stained for intracellular markers APC anti-Arginase 1 (369706, Biolegend, USA); PE anti-iNOS (696806, Biolegend, USA) with a Foxp3/Transcription Factor Staining Buffer Set (00-5523-00, eBioscience). Dead cells were excluded using a Fixable Viability Dye eFluor 455 (Thermo). Macrophages were identified with a CD45^+^CD11b^+^ gate. Flow cytometry was performed with a Cytek Aurora instrument, and data were analyzed with FlowJo software (version 10.6.2).

### scRNA-seq and data processing

#### Preparation of a single-cell suspension

Mice kidneys were harvested and digested using Multi Tissue dissociation kit (Miltenyi, 130-110-201). The tissue was homogenized using 21 G and 26.5 G syringes. Supernatants of the digested samples were collected in RPMI with 10% FBS on ice, followed by meshing through a 40 μm cell strainer with the syringe head. The total digested renal single cells were centrifuged at 600 g at 4 °C for 5 min, followed by red blood cell lysis. Cell number and viability were analyzed using Countess AutoCounter (Invitrogen, C10227).

#### cDNA Library Preparation and scRNA-seq

Kidney single cell suspension with qualified viability was counted and loaded for 10× Chromium scRNA-seq Library preparation and Illumina sequencing (LC-Bio Technology). Kidney cells were loaded onto the Chromium Next GEM Chip G for each group, and encapsulated into Gel in-beads emulsions (GEM) by the 10× Chromium Controller. Upon dissolution of the Single Cell 5’ Gel Bead in a GEM, oligonucleotides are released and mixed with cell lysate and a Master Mix that contains reverse transcription (RT) reagents and poly(dT) primers. Incubation of the GEMs then produces barcoded, full-length cDNA from poly-adenylated mRNA. After cDNA amplification, 5’ gene expression libraries are established. The final libraries were sequenced on NovaSeq 6000 (Illumina) with 150 bp paired-end reads.

#### Quality control of scRNA-seq data

Raw sequencing data were converted to fastq files with the Illumina bcl2fastq tool. Then, the 10× Genomics CellRanger pipeline was used to export gene expression matrix for each group, as well as an additional application of the CellRanger module aggr function to aggregate libraries from control, AKI, and AKI-P1705434 groups for comparative analysis. The raw scRNA-seq data were preprocessed using the Cell Ranger Single Cell Software Suite (V.5.0.1) provided by 10x Genomics for demultiplexing cellular barcodes, read alignment, and generation of the gene-cell matrix. Detailed QC metrics were generated and evaluated by the Seurat R package (V.4.0.5). Genes detected in less than 3 cells and cells in which detected transcripts were fewer than 200 or more than 8000 genes, or >70% UMIs derived from mitochondrial genes, or log10 gene count/log10 UMI count >0.80 were filtered out and excluded from the subsequent analysis.

#### Cell clustering and annotation

Seurat R package (V.4.0.5) was applied to filtered gene-cell count matrix normalization, scaling, and highly variable genes identification with default parameters. The optimal number of principal components (PCs) was determined by the ElbowPlot function. The top 2000 variable genes and the first 20 PCs selected by Seurat were used for unsupervised clustering analysis with a resolution set to 0.1. Cell-type identification was based on canonical cell-type-specific markers. For visualization, the dimensionality was reduced using UMAP methods with the RunUMAP function of Seurat. Cluster-specific marker genes were identified via the FindAllMarkers function with the following criteria: only.pos=TRUE; min.pct=0.25; and log FC ≥ 0.25.

#### Functional Enrichment Analysis

PT and CD cells functionally related gene set enrichment was tested by the GSVA R package (version 1.42.0) within a panel of annotated gene databases (Kyoto Encyclopedia of Genes and Genomes: http://software.broadinstitute.org/gsea/msigdb/genesets.jsp?collection=CP:KEGG, Human Immunosuppression Gene Atlas: http://biokb.ncpsb.org/HisgAtlas).

#### SCENIC analysis

SCENIC (V.1.3.1) was used to analyze activated regulons in cell clusters with a raw count matrix as input. The coexpression network was calculated by GRNBoost2, and the regulons were identified by RcisTarget. The regulon activity for each cell was scored using AUCell (V.1.16.0). Two-tailed Wilcoxon rank-sum tests were used to identify differentially activated regulons. Benjamini-Hochberg procedure was used to correct multiple hypotheses. The significant TFs were identified as adjusted p < 0.01, and top TFs across subclusters were determined according to log2 FC.

#### Cell Development Trajectories Construction

Single-cell pseudotime trajectories of CD cell populations were reconstructed by the Monocle 2 (version 2.22.0) package. Genes used for pseudotime ordering were taken from DEGs identified by function differentialGeneTest with fullModelFormulaStri set. Dimension reduction and cell ordering along the trajectories were completed with the DDRTree method.

### ROS assay kit

Cells were subjected to different treatments, and the ROS levels of different groups of cells were detected using the ROS assay kit (S0033S, Beyotime). Experiments were performed according to the manufacturer’s instructions.

### OCR

The rate of oxygen consumption (OCR) was measured with a Seahorse XF96 extracellular analyzer (Agilent). Briefly, 1×10^5^ mCCDcl1 cells were seeded on Seahorse XF96 culture plates in Seahorse XF DMEM Medium containing 1 mM pyruvate, 2 mM glutamine, and 10 mM glucose. OCR was measured by adding inhibitors at the indicated times with the following concentrations: oligomycin (1.5 mM), FCCP (2 mM), Antimycin A/Rotenone (0.5 mM). Three readings were taken after each injection, and the results were presented with pmoles/min for OCR (y axis) versus time (x axis). All experiments were performed at least three times.

### Mitochondrial complex IV activity assay

Kidney tissues obtained from mouse models treated with cisplatin or P1705434 and assayed for Mitochondrial complex IV Activity using Mitochondrial Complex IV / Cytochrome C Oxidase Activity Assay Kit (BC0940, Solarbio). Briefly, cells were lysed by the extract to obtain a precipitate. The cell precipitate was further extracted and fragmented for activity assay and protein content determination. All experiments were performed at least three times. Experiments were performed according to the manufacturer’s instructions.

### JC-1 assay

mCCDcl1 cells were subjected to different treatments, and the mitochondrial cell membrane potential levels of different groups of cells were detected using the JC-1 assay kit (C2006, Beyotime). Experiments were performed according to the manufacturer’s instructions.

### Renal histology, immunohistochemistry (IHC), and blood analysis

All paraffin-embedded mouse kidney sections (5 μm stick) were deparaffinized and blocked. Collagen deposition in renal tissue was evaluated by Masson’s trichrome staining (MTS, HT15, Sigma, USA) according to the manufacturer’s instructions. For immunohistochemistry (IHC), tissue sections were incubated with primary antibodies against F4/80 (D2S9R, CST, USA), Vimentin (ab92547, Abcam, UK), E-Cadherin (20874-1-AP, Proteintech), and α-SMA (14395-1-AP, Proteintech). Primary antibodies were diluted in bovine serum albumin and applied overnight at 4 °C in a wet chamber. After incubation in secondary antibodies, the diaminobenzidine reagent was added to these tumor sections, which were then counterstained with haematoxylin to visualize nuclei. We performed quantitative analysis of the corresponding markers by calculating the proportion of positive cells in the field of view to all cells using ImageJ (http://www.imagej.nih. gov/ij).

### RT and real-time PCR

RNA was reverse transcribed using 5 x Evo M-MLV RT Master Mix (AG11706, Acurrate Biology). qPCR was conducted with 2 x SYBR® Green Pro Taq HS Premix (Rox Plus) (AG11706, Acurrate Biology) in a QuantStudio 5 system (Applied Biosystems). Information about the primers is summarized:

KIM1-forward: 5’-CAGGGAAGCCGCAGAAAA-3’, reverse:5’-GAGACACGGAAGGCAACCAC-3’; TNF-forward: 5’-CCGATGGGTTGTACCTTGTC-3’, reverse:5’-GGCAGAGAGGAGGTTGACTTT-3’; CCL2-forward: 5’-CTCTTCCTCCACCACCAT-3’, reverse:5’-CTCTCCAGCCTACTCATTG-3’; β-actin-forward: 5’-CATTGCTGACAGGATGCAGAA-3’, reverse: 5’-ATGGTGCTAGGAGCCAGAGC-3’.

### Immunoblotting

Proteins were extracted using RIPA buffer (P0013B, Beyotime) containing a protease inhibitor and were quantified with a BCA kit (Thermo Fisher Scientific). Cell or tissue lysates containing equal amounts of protein were loaded in each well of a 10–17% SDS–PAGE gel. After electrophoresis, membrane transfer, and blocking, membranes were incubated with the indicated primary antibodies and HRP-conjugated secondary antibodies (31430, 31460, Invitrogen). The bands were visualized by enhanced chemiluminescence using Clarity™ Western ECL Substrate (Bio-Rad). The following antibodies were used: HIF-1α (20960-1-AP, Proteintech), HIF-2α (ab199, Abcam, UK), Smad3 (ab40854, Abcam, UK), VHL (ab77262, Abcam, UK), α-Tubulin (11H10, CST, USA), α-SMA (14395-1-AP, Proteintech), and KIM-1 (ab47635, Abcam, UK).

## Results

### Upregulation of Smad3 and imbalance of HIF-α were observed in the kidneys

**Fig. 1:**
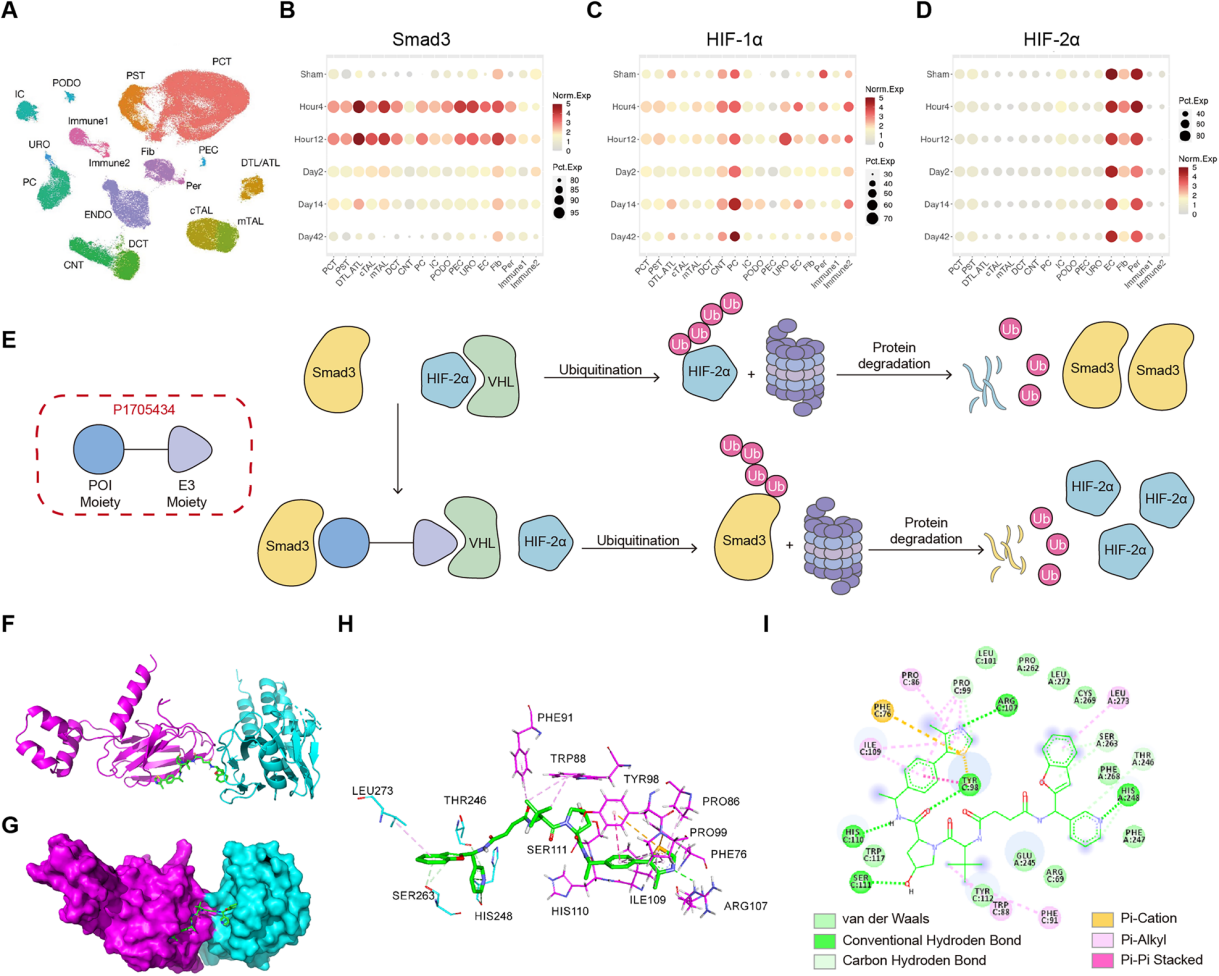
Upregulation of Smad3 and imbalance of HIF-α were observed in the kidneys. **A** Uniform manifold approximation and projection (UMAP) plot from mouse kidneys after bilateral IRI based on single-nuclear RNA sequencing, showing 16 subclusters of KIT database (http://humphreyslab.com/SingleCell/). PT, proximal tubule; PCT, proximal convoluted tubule; PST, proximal straight tubule; PEC, parietal epithelial cells; DTL/ATL, descending/ascending thin limb of Henle’ s loop; cTAL/mTAL; cortical/medullary thick ascending limb of Henle’ s loop; DCT, distal convoluted tubule; CNT, connecting tubule; PC, principal cells; ICA, type A intercalated cells; ICB, type B intercalated cells; PODO, podocytes; ENDO, endothelial cells; FIB, fibroblasts; Per, pericytes; Immune, immune cells; URO, uroepithelium. **B** The expression of Smad3, **C** HIF-1α and **D** HIF-2α along the time course in each subcluster in bilateral IRI mouse kidneys. **E** P1705434 recruits VHL to degrade Smad3 and meanwhile stabilizes HIF-2α level. **F** Cartoon and **G** surface representation of VHL (cyan) binding with P1705434 (green) sequence of Smad3 (magenta). The 3D (**H**) and 2D (**I**) interactions of P1705434 (green) molecule binding to the VHL and Smad3 protein complex. Line and stick models show the binding pocket structure of P1705434 with VHL and Smad3.

Aberrant expression of Smad3 and imbalanced expression of HIF-1α and HIF-2α are important factors in the AKI-CKD transition. We first performed analyses on the expression levels of Smad3, HIF-1α and HIF-2α in mouse ischemia-reperfusion injury (IRI) single-cell RNA sequencing (scRNA-seq) from Kidney Interactive Transcriptomics (KIT) database (http://humphreyslab.com/SingleCell/) [13, 14] (Fig. 1A). The results revealed a significant increase in Smad3 levels in early stage of AKI at hour 4 and hour 12, observed in most cell types (Fig. 1B). Increased expression of HIF-1α (Fig. 1C) at hour 4 and hour 12 was also noted in proximal tubular cells. Change of HIF-2α expression was not obvious (Fig. 1D) at AKI stages. To degrade excessive Smad3 accumulation and improve the imbalance between HIF-1α and HIF-2α, we designed a Smad3-targeted PROTAC named P1705434 (Fig. 1E). P1705434 was constructed by linking the von Hippel-Lindau (VHL) recognition ligand and the Smad3 recognition ligand together, which targets the Smad3 protein to degradation via the ubiquitin-proteasome pathway (UPP). The degradation of Smad3 is mediated through the proteasome by hijacking the activity of E3 ubiquitin ligases VHL to ubiquitinate Smad3. Under physiological state, VHL mediates the degradation of HIF-2α via UPP. With the application of P1705434, VHL will mediate the degradation of Smad3 instead of HIF-2α. To provide structural insight into the dual-target of P1705434, we performed molecular docking to model the ternary complex VHL–P1705434–Smad3 (Fig. 1F, G). The model demonstrates that P1705434 simultaneously engages both proteins through specific interactions (Fig. 1H). Analysis of the binding interfaces revealed key stabilizing interactions: a hydrogen bond and π-alkyl with VHL, and multiple hydrogen bonds alongside a π-cation interaction with Smad3, all supported by extensive complementary van der Waals forces (Fig. 1I). This structural model validates the rational design of P1705434 and illustrates the molecular basis for its ability to recruit Smad3 to VHL for ubiquitination. We next sought to validate this degradation mechanism in a cellular model relevant to AKI. We tested the protein expression level in the HK2 cell line treated by cisplatin (Supplementary Fig. S1A). HIF-1α increased after cisplatin treatment, but didn’t decrease after P1705434 treatment (Supplementary Fig. S1B). HIF-2α was accumulated, and Smad3 was reduced significantly after treatment with P1705434 (Supplementary Fig. S1C, D). This result proved that P1705434’s targeted proteins were Smad3 and HIF-2α.

### P1705434 ameliorated renal function loss in cisplatin-induced nephrotoxicity mice

**Fig. 2:**
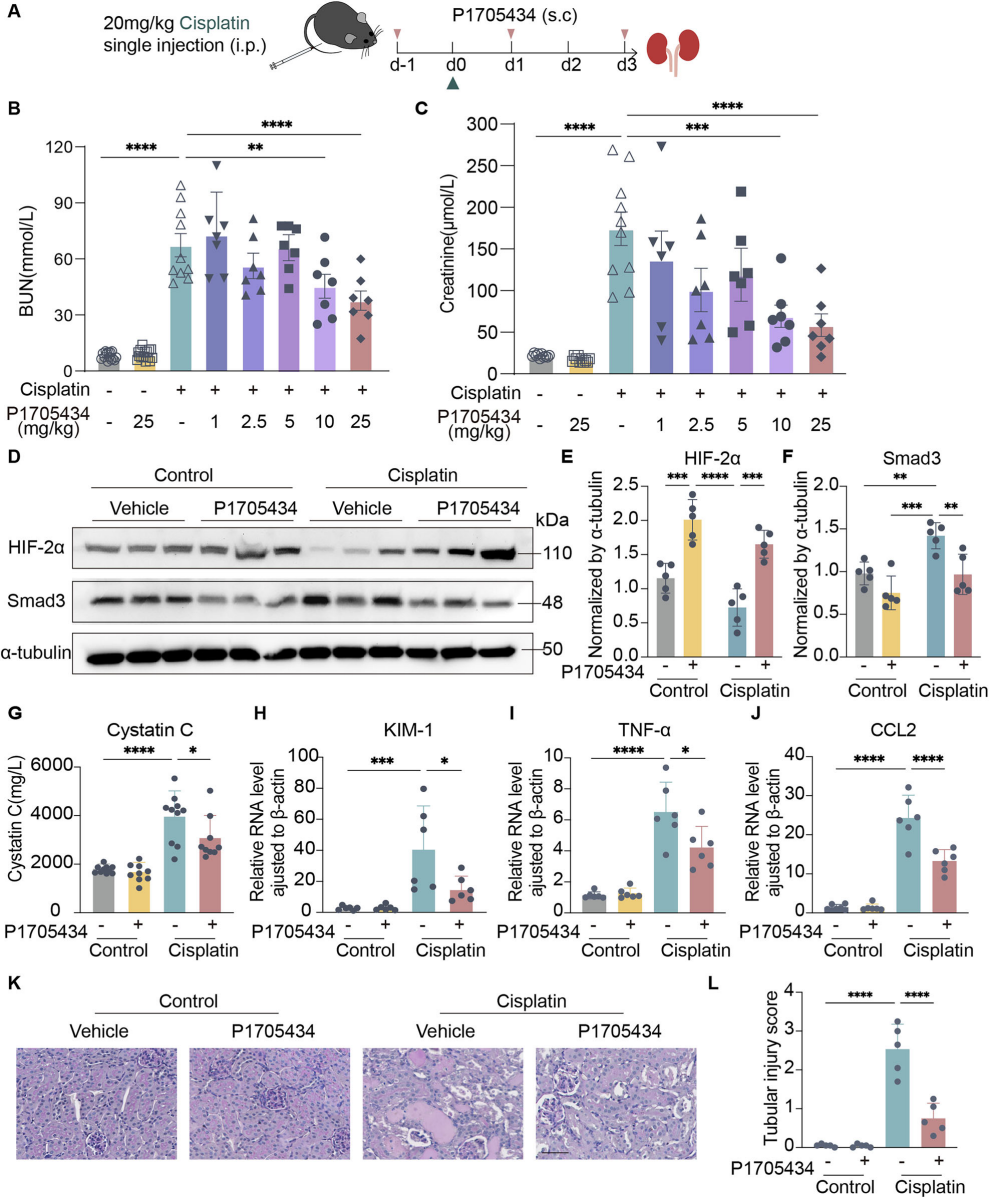
P1705434 ameliorates renal function loss in cisplatin nephrotoxicity mice. **A** Experimental design of evaluating the therapeutic effect of P1705434 on cisplatin nephrotoxicity model. Renal function was evaluated with **B** BUN and **C** serum creatinine at indicated dose of P1705434. **D** Western blot analyses of HIF-2α and Smad3 expression and (**E**, **F**) quantification of them in cisplatin mice (*n* = 5). **G** Cystatin C level of mice treated with P1705434 in cisplatin nephrotoxicity model. **H** Transcriptional level of KIM-1, **I** TNF-α and **J** CCL2 in cisplatin nephrotoxicity model. **K** Representative Periodic Acid-Schiff (PAS) staining image in P1705434 untreated or treated mice following cisplatin or saline injection. **L** Semi-quantitative analysis of tubular injury score in PAS staining of **K** (*n* = 5). Data are means ± SD, *P < 0.05, **P < 0.01, ***P < 0.001, ****P < 0.0001.

To further validate the early protective effects and mechanisms of P1705434 on AKI, the widely accepted cisplatin-induced nephrotoxicity mouse model was employed. P1705434 was administered one day prior to modeling and subsequently every other day (Fig. 2A). Minimum effective concentration was first detected by the administered concentration gradient of P1705434. Results of blood urea nitrogen (BUN) and serum creatinine showed that cisplatin-induced decline in renal function can be significantly ameliorated by P1705434 when the concentration reached 10 mg/kg and 25 mg/kg (Fig. 2B, C). Immunoblotting of mouse kidneys revealed a reduction in Smad3 expression and an increase in HIF-2α expression in P1705434-treated mice of both control and cisplatin groups (Fig. 2D–F). Serum Cystatin C levels were also measured, which were significantly elevated following cisplatin injection but were reduced by P1705434 administration (Fig. 2G). RNA expression level of kidney injury marker kidney injury molecule-1 (KIM-1) and inflammation marker Tumor Necrosis Factor-alpha (TNF-α) and C-C Motif Chemokine Ligand 2 (CCL2) were all significantly elevated in the cisplatin-vehicle group and reduced after P1705434 treatment (Fig. 2H–J). P1705434-treated cisplatin-induced AKI mice showed less cast formation, tubular dilatation, and epithelial cell atrophy, with a lower tubular injury score (Fig. 2K, L). These findings indicate the protective role of P1705434 in cisplatin-induced nephrotoxicity in mouse kidneys.

### P1705434 ameliorated FAN mice renal function loss by decreasing Smad3 and stabilizing HIF-2α

**Fig. 3:**
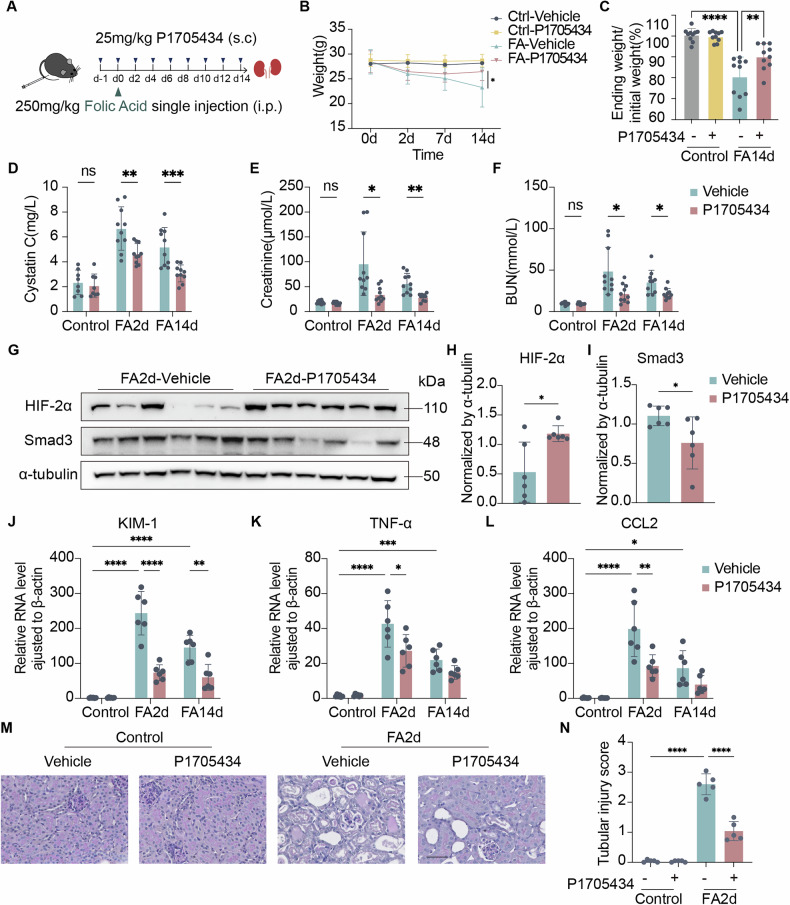
P1705434 ameliorates renal function loss in FAN mice. **A** Experimental design of evaluating the therapeutic effect of P1705434 on FAN model. **B** Trajectory of weight of indicated groups at indicated times (*n* = 10). **C** Weight changes of indicated groups from initial to ending. **D** Trajectory of renal function was evaluated with Cystatin **C**, **E** serum creatinine, and **F** BUN at indicated times (*n* = 10). **G** Western blot analyses of HIF-2α and Smad3 expression and (**H**, **I**) quantification of them in FA2d mice (*n* = 6). **J** Transcriptional level of KIM-1, **K** TNF-α and **L** CCL2 in cisplatin nephrotoxicity model. **M** Representative Periodic Acid-Schiff (PAS) staining image in P1705434 untreated or treated mice of FA2d mice kidneys. **N** Semi-quantitative analysis of tubular injury score in PAS staining of (**N**) (*n* = 6). i.p. = intraperitoneal, FA = folic acid. Data are means ± SD, ns = not significant, *P < 0.05, **P < 0.01, ***P < 0.001, ****P < 0.0001.

To evaluate the role of P1705434 in the AKI-CKD transition in mice, the folic acid nephropathy (FAN) model was selected (Fig. 3A). Over the 14-day period of the FAN model, body weights in the FA-P1705434 group exhibited a slight reduction followed by a rebound, whereas the FA-Vehicle group showed a continuous decline (Fig. 3B, C). Renal function was assessed by measuring serum Cystatin C, creatinine, and blood urea nitrogen (BUN), all of which were significantly elevated following FA injection. Creatinine and BUN levels peaked at day 2 and subsequently decreased slightly by day 14. Compared to the FA-Vehicle group, renal function in the FA-P1705434 group was significantly impaired at both day 2 and day 14 (Fig. 3D–F), but at day 28, Cystatin C, creatinine, and BUN levels in all groups had nearly returned to baseline, showing no significant differences between treatment groups (Supplementary Fig. S2C, E, G). Immunoblotting revealed that Smad3 levels were reduced and HIF-2α levels were increased following P1705434 treatment at FA2d (Fig. 3G–I). KIM-1, TNF-α and CCL2 expression were all significantly elevated in FA2d and reduced in P1705434 treatment (Fig. 3J–L). PAS staining showed P1705434-treated FA2d AKI kidneys had lower tubular injury (Fig. 3M, N). These results indicated that reducing renal Smad3 and stabilizing HIF-2α expression by applying P1705434 could protect the renal function in FAN mice, and the protective role was exerted at an early stage during AKI-CKD transition.

### P1705434 attenuated kidney fibrosis and inflammation in FAN mice

**Fig. 4:**
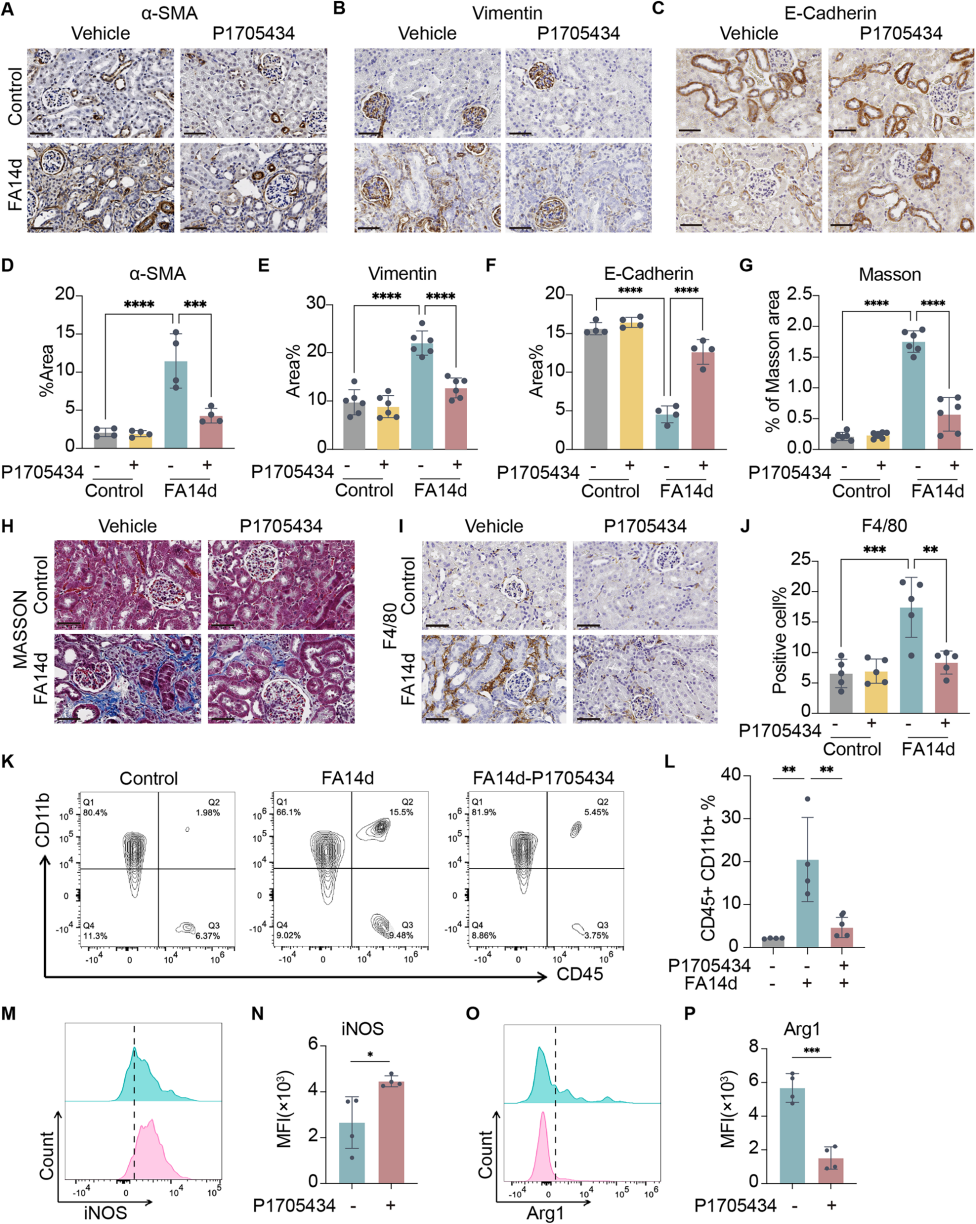
P1705434 attenuates kidney fibrosis and inflammation of FAN mice. **A** Representative IHC staining image of α-SMA, **B** Vimentin, and **C** E-Cadherin among the indicated groups. **D** The positive area ratio of α-SMA (*n* = 4), **E** Vimentin (*n* = 6) and **F** E-Cadherin of the indicated groups (*n* = 4). **G** The positive area ratio of Masson’ s trichrome staining of the indicated groups and **H** representative staining image. **I** Representative F4/80 IHC staining of renal sections among the indicated groups. **J** The count of F4/80^+^ macrophages/HPF of indicated groups (*n* = 5). **K** Flow-cytometry analysis of the kidney cell population in CD45^+^ CD11b^+^ of indicated groups (*n* = 4). **L** Quantification of the kidney-infiltrating immune cells (CD11^+^CD45^+^) percentage in indicated groups (*n* = 4). **M** Representative image and histogram, and **N** quantification of the expression of iNOS in macrophages (*n* = 4). **O** Representative image and histogram and (**P**) quantification of the expression of Arg1 in macrophages (*n* = 4). FA = folic acid. Data are means ± SD, *P < 0.05, **P < 0.01, ***P < 0.001, ****P < 0.0001. Scale bars: 100 μm.

To determine whether P1705434 attenuates renal fibrosis in AKI-CKD transition, immunohistochemical (IHC) and Masson’s trichrome staining were performed on FAN mice. IHC staining revealed a marked induction of the fibrosis-associated marker α-smooth muscle actin (α-SMA) in vehicle-treated day 14 FAN mice, which was significantly reduced by P1705434 treatment (Fig. 4A, D). This suppression of α-SMA expression was sustained at the chronic stage of day 28 (Supplementary Fig. S2I, K). Similarly, the expression of Vimentin, another marker associated with fibroblast activation and epithelial-mesenchymal transition, was significantly elevated at day 14 in FAN kidneys and effectively suppressed by P1705434 (Fig. 4B, E). Conversely, expression of epithelial adhesion marker E-cadherin, which was diminished after FA injury, was preserved in P1705434-treated mice (Fig. 4C, F). MASSON staining showed that collagen deposition in the kidneys of the vehicle group was significantly reduced compared with the P1705434 group on day 14 (Fig. 4G, H), consistent results were also found in the day 28 outcome (Supplementary Fig. S2H, J). To assess whether P1705434 attenuates renal inflammation, the macrophage marker F4/80 was evaluated. Significantly higher macrophage infiltration was observed in the FAN kidneys compared to the control group, whereas macrophage infiltration was significantly reduced in the P1705434 group (Fig. 4I, J). Flow cytometry analysis of macrophages in mouse kidneys revealed a reduction observed in the FA14d-P1705434 group relative to the vehicle group (Fig. 4K, L). Additionally, macrophages in the FA14d-P1705434 group exhibited higher expression of the M1 marker inducible nitric oxide synthase (iNOS) (Fig. 4M, N), while the FA14d-Vehicle group showed elevated expression of the M2 marker arginase-1 (Arg1) (Fig. 4O, P). M1 macrophages are primarily pro-inflammatory, contributing to infection clearance but also promoting renal injury, while M2-type macrophages are anti-inflammatory, contributing to tissue repair but promoting renal fibrosis [13].

### Evaluation of P1705434 against established combination treatment strategies

To comprehensively evaluate the potential advantages of the PROTAC strategy, we directly compared P1705434 with established therapeutic approaches both in vivo and in vitro. FAN Mice were treated with the vehicle, P1705434, or a combination of Smad3 inhibitor SIS3 and HIF-PHD inhibitor FG-4592 (Roxadustat) (Supplementary Fig. S2A). Assessment of renal function at the acute phase (day 2) revealed that FA injection induced significant increases in serum Cystatin C, creatinine, and BUN. Both P1705434 treatment and the SIS3 + FG-4592 combination therapy significantly ameliorated this acute renal dysfunction to a similar extent (Supplementary Fig. S2B, D, F). By the chronic phase (day 28), serum levels of Cystatin C, creatinine, and BUN in all groups, including the vehicle-treated FAN mice, had largely returned to baseline, showing no significant differences between treatment groups (Supplementary Fig. S2C, E, G). In contrast, Masson’s trichrome staining showed pronounced collagen deposition in vehicle-treated FAN mice, which was markedly attenuated by both P1705434 and the SIS3 + FG-4592 combination (Supplementary Fig. S2H, J). Induction of α-SMA in FAN kidneys was significantly and comparably reduced by both treatments (Supplementary Fig. S2I, K).

PROTACs exert their effects through a catalytic, event-driven mechanism, which can confer a more durable pharmacological response compared to traditional occupancy-driven inhibitors. To directly compare the duration of action between P1705434 and the inhibitor combination, we performed in vitro wash-out experiments. TGF-β-stimulated HK2 cells were treated with P1705434 or the SIS3 + FG-4592 combination for 24 h. Following drug removal and replacement with fresh medium, cells were cultured for an additional 24 or 48 h before analysis. Immunoblotting results showed cells treated with P1705434 maintained significantly elevated HIF-2α levels and reduced Smad3 protein levels even at 48 h after wash-out (Supplementary Fig. S2L-N).

Collectively, these comparative studies indicate that P1705434 not only achieves an anti-fibrosis effect equivalent to a combination of two inhibitors but also exhibits a more sustained mode of action due to its catalytic degradation mechanism, highlighting its potential as an AKI-CKD transition therapeutic strategy.

### Cisplatin-induced AKI single-cell RNA-seq exhibited cell heterogeneity

**Fig. 5:**
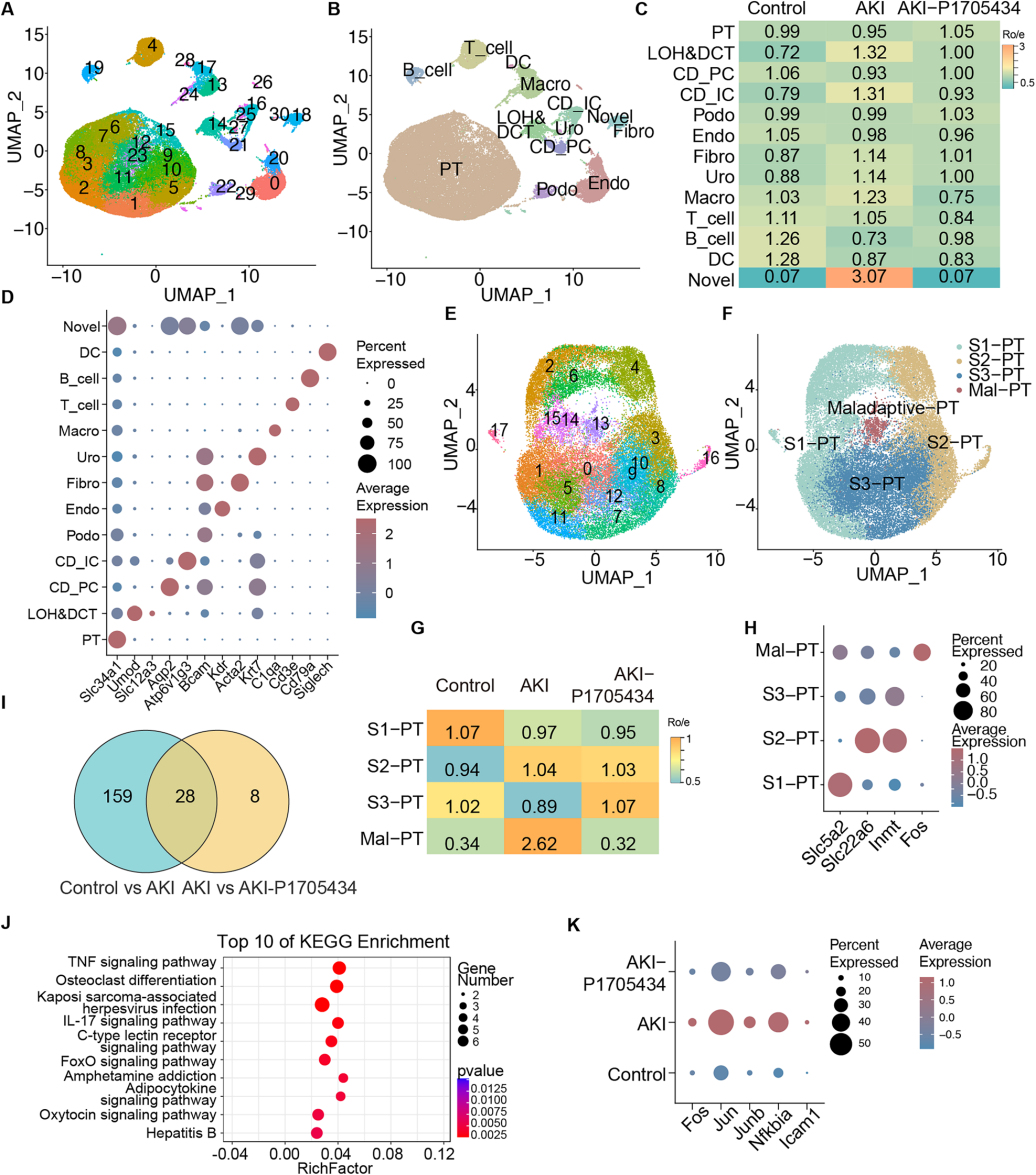
Single-cell RNA sequencing of renal cells identifies 13 distinct cell subpopulations in mice with cisplatin nephrotoxicity. **A** UMAP plots of all mouse cisplatin nephrotoxicity kidney datasets integrated with Harmony and **B** divided into 13 clusters. PT: proximal tubule; LOH: loop of Henle; DCT: distal convoluted tubule; CD-IC: CD intercalated cell; CD-PC: collecting duct principal cell; Podo: podocytes; Endo: endothelial; Fib: fibroblasts; Uro: urothelial cell; Macro: macrophage; DC: dendritic cell. **C** Heatmap displaying composition of clusters by groups, and **D** dot plot showing gene expression level of cell specific markers. **E** UMAP displaying the clustering of proximal tubular cells and **F** divided into 4 clusters: S1: S1 segment of proximal tubule; S2: S2 segment of proximal tubule; S3: S3 segment of proximal tubule. Mal-PT: maladaptive proximal tubule. **G** Heatmap displaying composition of proximal tubular cells subclusters by groups, and **H** dot plot showing gene expression level of cell specific markers. **I** Venn diagram showing the overlaps of differentially expressed genes in S3-PT cells between the Control vs AKI and AKI vs AKI-P1705434 groups. **J** Enrichment ratios of top 10 enriched KEGG pathway terms, derived by overrepresentation analysis of the overlap DEGs of **I**. **K** Dot plots showing expression of genes in PT cells involved in TNF signaling pathway. PCT percentage of cells expression.

Given the heterogeneity of kidney cells, scRNA-seq was performed on mouse kidneys to assess the effects of P1705434 treatment on AKI at the cellular level. Following quality control screening, 51,016 cells were isolated from mouse kidneys. The single-cell transcriptome was visualized using UMAP (Uniform Manifold Approximation and Projection) plots, which classified the cells into 13 distinct clusters: proximal tubular (PT), loop of Henle (LOH), distal convoluted tubule (DCT), collecting duct intercalated cells (CD-IC), collecting duct principal cells (CD-PC), podocytes (Podo), endothelial cells (Endo), fibroblasts (Fibro), urothelial cells (Uro), macrophages (Macro), T cells, B cells, dendritic cells (DC), and a novel cell population (Fig. 5A, B). Chi-square analysis of the ratio of observed to expected cell numbers (Ro/e) in each cluster revealed significant differences in cell type distribution among the three kidney groups (Fig. 5C). Major cell types and subclusters were identified based on cell type-specific markers (Fig. 5D).

### P1705434 reduced proximal tubular cell injury in AKI

Proximal tubular (PT) cells constitute the largest proportion of all cell types, and they are the major cell experienced AKI. To analyze changes in PT cells, PT cells were further reclustered into four subpopulations based on PT-specific marker genes: segment 1 (S1-PT), segment 2 (S2-PT), segment 3 (S3-PT), and maladaptive PT (Fig. 5E, F). Comparison of cell numbers across groups revealed a significant decrease in S3-PT cells in the AKI group (Ro/e = 0.89), with improvement observed in the AKI-P1705434 group (Ro/e = 1.07). Additionally, a significant increase in maladaptive PT cells was noted in the AKI group (Ro/e = 2.62) (Fig. 5G). The expression of cell-specific markers in the four clusters is shown (Fig. 5H). Twenty-eight differentially expressed genes (DEGs) were identified in both the Control versus AKI and AKI versus AKI-P1705434 comparison groups (Fig. 5I). Further analysis of the overlapping DEGs in S3-PT cells from the two comparison groups revealed that the TNF signaling pathway was the most significantly enriched pathway (Fig. 5J). Genes enriched in the TNF signaling pathway, including Fos, Jun, Junb, Nfkbia, and Icam1, were significantly upregulated in the AKI group but showed improvement in the AKI-P1705434 group (Fig. 5K).

### P1705434 attenuated collecting duct cell transition to fibroblasts in AKI by preserving OXPHOS

**Fig. 6:**
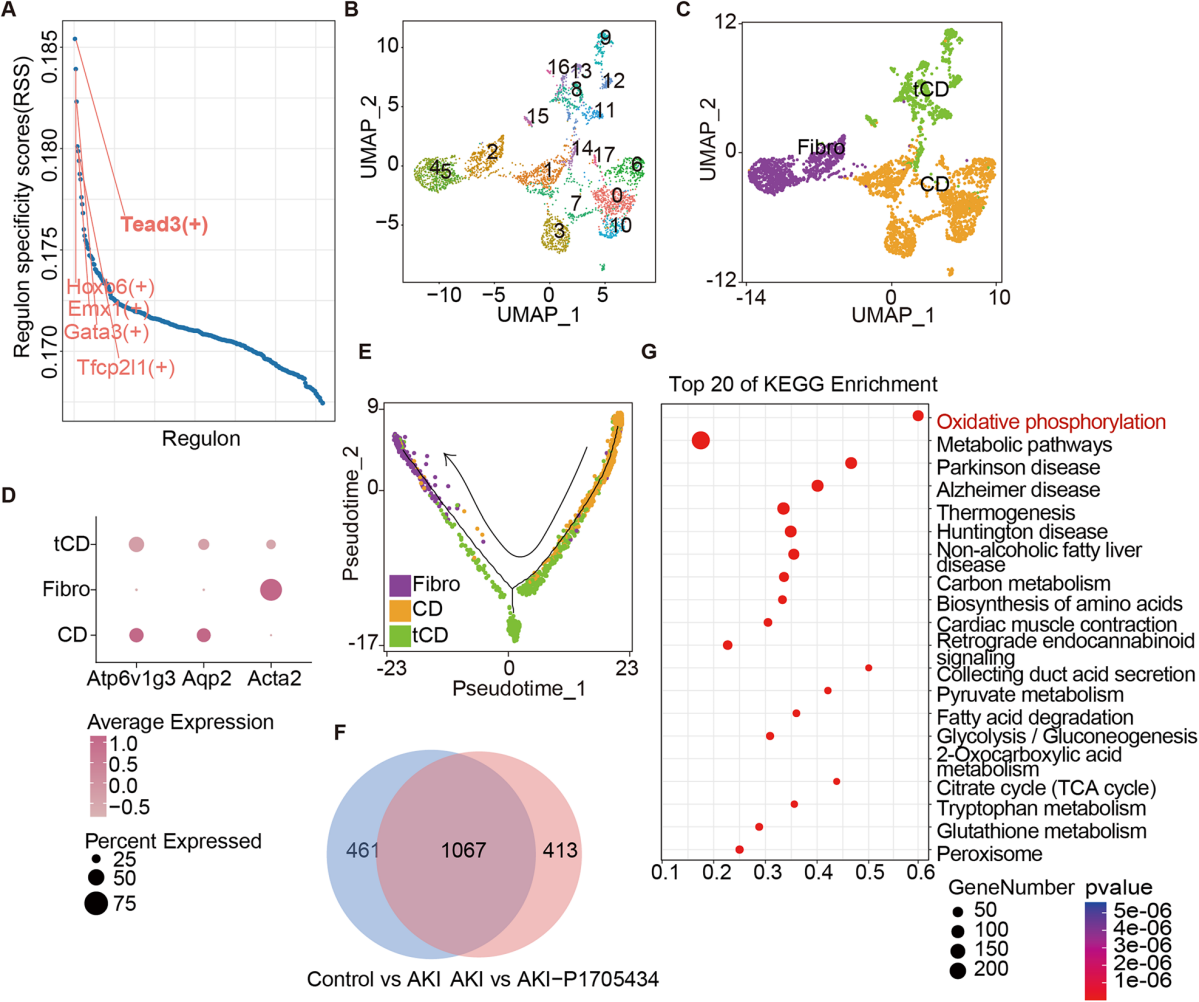
P1705434 attenuates the collecting duct cell transition to fibroblasts in cisplatin-induced AKI kidney. **A** SCENIC analysis showing the differential regulons of tCD cells. **B** UMAP displaying the clustering of CD cells and Fibroblasts and **C** divided into 3 clusters. Fibro: fibroblasts; CD: collecting duct cell; tCD: transitional collecting duct cell. **D** Dot plot showing gene expression level of cell-specific markers in (**B**). **E** The distribution of CD cells, tCD cells, and fibroblast clusters in a pseudo-time trajectory. **F** Venn diagram showing the overlaps of differentially expressed genes in CD cells between the Control vs AKI and AKI vs AKI-P1705434 groups. **G** Enrichment ratios of the top 20 enriched KEGG pathway terms, derived by overrepresentation analysis of the overlap DEGs of (**F**). Data are means ± SD, **P < 0.01, ****P < 0.0001.

A previously unidentified cell population, we termed transitional collecting duct (tCD) cells in this paper, was identified in Fig. 5B, exhibiting a high proportion (Ro/e > 3) in the AKI group (Fig. 5C). These tCD cells expressed marker genes for both intercalated cells (CD-IC; Atp6v1g3) and principal cells (CD-PC; Aqp2). SCENIC analysis revealed that tCD cells expressed the CD-IC-related transcription factor Tfcp2l1 (+), the CD-PC-related transcription factors Hoxb9 (+) and Gata3 (+), and the fibrosis-associated transcription factor Tead3 (+) (Fig. 6A). To investigate the relationship and mechanisms underlying this cluster, tCD cells, CD cells, and fibroblasts (Fibro) were reclustered (Fig. 6B, C). Figure 6D illustrated the expression of cell-specific markers in the three clusters. Combined with cell trajectory Integration of CytoTRACE cell trajectory analysis and pseudotime analysis suggested that CD cells may differentiate into fibroblasts, with tCD cells serving as intermediate transitional cells (Fig. 6E, Supplementary Fig. S3). Given the greater plasticity of CD-IC cells demonstrated in previous studies, CD-IC cells were selected for further analysis. Venn diagrams identified 1,067 DEGs in CD-IC cells from the Control versus AKI and AKI versus AKI-P1705434 comparison groups (Fig. 6F). Enrichment analysis revealed that the oxidative phosphorylation (OXPHOS) pathway was the most significantly enriched pathway among CD-IC DEGs (Fig. 6G). These results suggest that P1705434 treatment reduces the propensity of CD cells to differentiate into fibroblasts under AKI conditions.

### P1705434 protected mitochondrial function in AKI mice

**Fig. 7:**
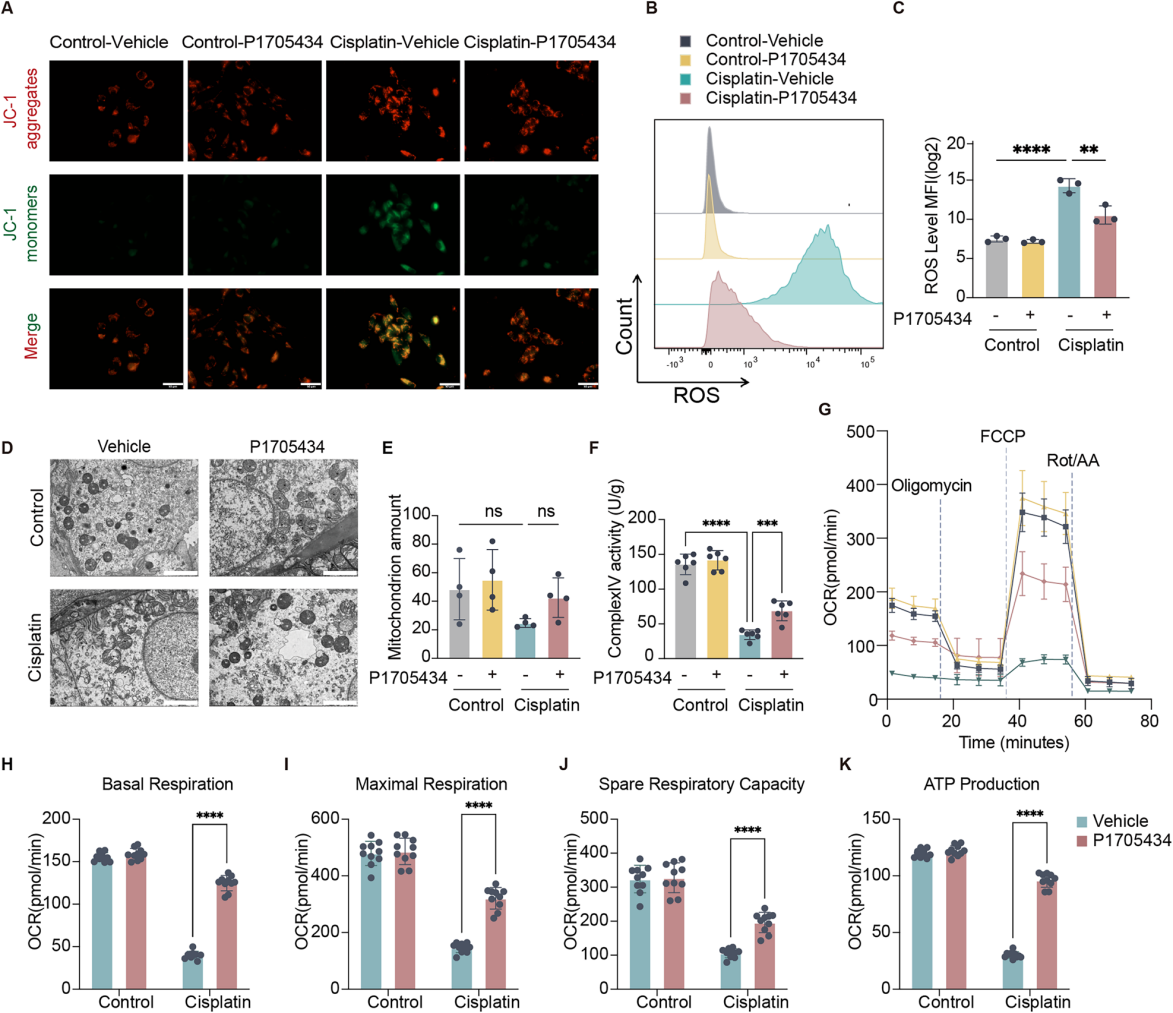
P1705434 promotes mitochondria function in cisplatin induced AKI. **A** Detection of mitochondrial membrane potential (MMP, ΔΨm) of primary collecting duct epithelium cells by using JC-1 agent. Scale bars: 50 μm. **B** Flow cytometry analysis to detect the changes in ROS accumulation of primary collecting duct epithelium cells and independent experiment result of **C** (*n* = 3). **D** Electron microscopy was performed on mice collecting duct and **E** mitochondrial counts was assessed. Scale bars: 500 nm. **F** Activities of mitochondrial complex IV of mice kidney tissue (*n* = 6). **G** Analyses of oxygen consumption rate (OCR) of collecting duct cells (*n* = 8) by using Seahorse extracellular flux analyzer. **H** Basal respiration, **I** maximal respiration, **J** spare respiratory capacity and **K** ATP production calculated by **G**. Data are means ± SD, **P < 0.01, ****P < 0.0001. JC-1, 5,5’,6,6’-tetrachloro-1,1’,3,3’-tetraethylbenzimidazolocarbocyanine iodide; ROS, reactive oxygen species.

To further validate the effect of P1705434 on the OXPHOS pathway in collecting duct cells, mitochondrial function was assessed in collecting duct epithelial cells. JC-1 assays revealed that the Cisplatin-Vehicle group exhibited weaker red fluorescence and stronger green fluorescence compared to the control group, indicating impaired JC-1 aggregation in the mitochondrial matrix and reduced mitochondrial membrane potential. Compared to the vehicle group, reduced green fluorescence was observed in the Cisplatin-P1705434 group, suggesting that P1705434 ameliorated the decline in mitochondrial membrane potential in collecting duct cells (Fig. 7A). The level of ROS production in collecting duct epithelial cells was significantly elevated in the Cisplatin-Vehicle group but improved in the Cisplatin-P1705434 group (Fig. 7B, C). Observation of the mitochondrial status of mouse collecting duct cells under electron microscopy revealed swollen and ruptured mitochondria in the cisplatin model mice, with more improved mitochondria in the P1705434 treatment group (Fig. 7D). However, the number of mitochondria did not differ significantly among the four groups (Fig. 7E). Electron transport chain (ETC) complex activity was measured in mouse kidney tissue extracts. Complex IV activity was significantly reduced in the cisplatin-induced nephrotoxicity model mice, and P1705434 ameliorated this reduction (Fig. 7F). Mitochondrial oxygen consumption rate (OCR) decreased following cisplatin treatment but showed significant improvement with P1705434 administration (Fig. 7G). Analysis of mitochondrial respiration parameters, including basal respiration, maximal respiration, spare respiratory capacity, and ATP production, further demonstrated the mitochondrial protective effects of P1705434 (Fig. 7H–K). Together, these results indicated that P1705434 treatment protected mitochondrial function of AKI mice.

## Discussion

P1705434 plays a role in protecting the kidneys in AKI-CKD transition by degrading Smad3 and stabilizing HIF-2α, which was validated in both FAN and cisplatin nephrotoxicity model mice. scRNA-seq analysis revealed that P1705434 reduced the proportion of maladaptive PT cells and downregulated the TNF pathway, thereby ameliorating tubular cell injury in S3-PT. Additionally, CD cells were identified as targets of P1705434 treatment, which reduced mitochondrial injury in collecting duct cells by upregulating the OXPHOS pathway and inhibited the transition of CD cells to fibroblasts, ultimately ameliorating renal fibrosis and inflammation.

Smad3 has been shown to be one of the most important signaling proteins in renal fibrosis, mediating not only the fibrotic effects of TGF-β1 but also contributing to the pro-fibrotic effects of angiotensin II (Ang II), hyperglycemia, advanced glycosylation end products (AGEs), and HIF-1α [15]. Numerous studies have demonstrated that inhibiting the Smad3 signaling pathway prevents renal fibrosis through various mechanisms. For instance, the Smad3 inhibitor SIS3 reduces renal fibrosis in a mouse model of diabetic nephropathy [16], and Smad3 knockout inhibits renal fibrosis in a unilateral ureteral obstruction (UUO) mouse model [17]. The degradation of abnormally upregulated Smad3 by the dual-target PROTAC P1705434 not only inhibits the overactive Smad3 signaling pathway but also represents an ideal therapeutic strategy, as this degradation is transiently reversible and preserves other essential physiological functions of Smad3, such as immune regulation.

Additionally, the imbalance of HIF-α isoforms plays a significant role in the progression of AKI to CKD. Stabilization of endothelial HIF-2α, but not HIF-1α, has been shown to prevent hypoxia-induced renal injury, highlighting HIF-2α as a potential therapeutic target for renal protection and fibrosis prevention following acute ischemic injury [18]. However, studies have also demonstrated that the PHD inhibitor FG-4592 protects against renal injury and attenuates renal fibrosis in AKI models by stabilizing HIF-α [19–21]. Since these studies did not assess the expression levels of individual HIF-α isoforms, and PHD inhibitors can theoretically stabilize both HIF-1α and HIF-2α, the precise mechanism underlying the renal protective effects of PHD inhibition remains unclear.

Our comparative analysis reveals a distinct pharmacological advantage of the PROTAC strategy over inhibitor combination therapy. While both P1705434 treatment and the SIS3 + FG-4592 combination achieved comparable therapy efficacy in AKI-CKD transition, P1705434 exhibited a more durable mode of action. Traditional inhibitors follow an occupancy-driven model, meaning they must continuously bind to a target’s active site at high concentrations to maintain inhibition, while PROTACs use an event-driven model, acting as catalysts that flag the protein for degradation by ubiquitin-proteasome [22]. PROTACs induced degradation provide a lasting “off-switch” effect for the targeted protein, but the inhibitors only temporarily block the targeted protein-related signaling pathway. Because the target protein is physically destroyed, its effect lasts until the cell can synthesize new proteins. Additionally, PROTACs can facilitate the degradation of multiple target protein molecules through repeated cycles of binding for ubiquitination and release, providing a more durable response even after the drug withdrawal [23].

The present study demonstrated reduced renal macrophage infiltration and a significant decrease in the proportion of M2-type macrophages in the dual-target PROTAC intervention group. Both Smad3 and HIF-α have been shown to promote macrophage activation and polarization (transition from M1 to M2 subtypes) [24]. Increased Smad3 expression and activation in progressive kidney disease not only directly promote myofibroblast differentiation and extracellular matrix (ECM) production but also modulate renal inflammation by enhancing macrophage polarization [25]. In contrast to the pro-inflammatory effects of HIF-1α, HIF-2α can inhibit macrophage activation by suppressing mitochondrial reactive oxygen species (ROS) [26]. HIF-1α has been shown to regulate inflammation by upregulating the M1 macrophage marker Arg1, while HIF-2α regulates inflammation by upregulating the M2 marker iNOS [27], consistent with our findings.

Cisplatin primarily damages the S3 segment of the proximal tubule [28]. The uneven partial pressure of oxygen in the renal cortex, combined with the high metabolic rate of proximal tubular segments S1 and S2, results in low oxygen pressure in the S3 segment, making S3-PT the primary site of hypoxic injury in the kidney [29]. The results of our study demonstrated that P1705434 treatment reduced the loss of proximal tubular cells in the S3 segment. KEGG enrichment analysis revealed that the intersection of the two comparison groups was primarily enriched in the TNF pathway. The dual-target PROTAC P1705434 mitigated the loss of proximal tubules in the S3 segment by downregulating Fos, Jun, Junb, Nfkbia, and Icam1. Notably, Fos, Jun, and Junb are downstream target genes of Smad3 and can form activator protein 1 (AP-1) [30]. AP-1, a transcription factor belonging to the Fos (c-Fos, FosB) and Jun (c-Jun, JunB, JunD) families, dimerizes through leucine zipper regions. Fos/Jun heterodimers, the predominant form of AP-1 in most cells, exhibit high binding affinity. It has been demonstrated that AP-1 can promote renal injury in renal tubular cells by facilitating the release of inflammatory factors and the recruitment of inflammatory cells [31].

Renal collecting duct epithelial cells consist primarily of principal cells (PC) and intercalated cells (IC). PCs regulate water and sodium re-absorption, while ICs maintain acid-base homeostasis. Our study identified a population of transition-state cells in the AKI group that expressed both IC and PC markers and exhibited potential to differentiate into fibroblasts. One study has demonstrated that mouse renal tissues contain transitional-state cells expressing both IC and PC markers, which promote renal fibrosis [32]. This is consistent with the tCD cell population identified in our study. ICs are highly plastic and strongly associated with renal tubular acidosis [32]. The kidney’s active reabsorptive and secretory functions necessitate high energy consumption, resulting in the highest resting metabolic rate of any organ except the heart. Consequently, mitochondrial function is critical for maintaining renal energy homeostasis [33]. Hypoxia limits substrate (O_2_) availability for Complex IV, slowing electron transfer in the electron transport chain (ETC) and reducing proton pumping, which generates the proton motive force (Δp), the electrochemical and proton concentration gradient, thereby reducing OXPHOS activity [34]. Under conditions of high metabolism, renal ATP consumption remains unchanged, while OXPHOS efficiency declines, leading to ATP deficiency and impaired renal energy supply. Additionally, reduced Complex IV activity results in aberrant electron transfer to molecular oxygen and excessive ROS production [26]. HIF-1α and HIF-2α regulate ROS production through distinct mechanisms. HIF-1α upregulates NADPH oxidase 2 (NOX2), increasing ROS production, while HIF-2α upregulates mitochondrial superoxide dismutase 2 (SOD2), which suppresses ROS production [26].

Widely used animal models of AKI include renal ischemia-reperfusion injury (IRI), folic acid nephropathy (FAN), and cisplatin-induced nephrotoxicity model [35]. Although IRI was historically the most widely used animal model for AKI research, it is not a common cause of clinical AKI [36]. In clinical settings, renal blood flow is rarely completely interrupted, except during renal transplantation or cross-clamping of the aorta or renal artery. Additionally, ischemic conditions in clinical procedures differ from those in animal models [37]. AKI is primarily caused by systemic hemodynamic changes, hypoxemia, and nephrotoxins. The FAN model is commonly used to simulate AKI and represents a typical model of the AKI-CKD transition. The mechanism of FAN involves high-dose folic acid injection, leading to the accumulation of folic acid crystals in renal tubules. These crystals cause nephrotoxicity through direct toxicity and physical damage [38]. Therefore, we constructed the FAN model and the cisplatin model to explore the role and mechanism of P1705434 on AKI instead of an IRI model.

### Statistical information

Experimental data are presented as the mean ± standard deviation (SD) of at least three biological replicates. *P* value<0.05 was considered statistically significant. The degree of significance between groups is represented as follows: *P < 0.05; **P < 0.01; and ***P < 0.001. Statistical tests were carried out with GraphPad Prism (version 8).

## Supplementary information

Supplementary figure

original western blots

## Data Availability

The scRNA-seq data have been deposited in the CNCB database under the accession ID PRJCA029666 (https://ngdc.cncb.ac.cn/).
