# Supplementary material for: VHL-recruiting PROTAC attenuates AKI-CKD transition via simultaneous degradation of Smad3 and stabilization of HIF-2α
Source: Cell Death Dis. 2026 Apr 10;17(1):460. doi: 10.1038/s41419-026-08726-w (PMC13181044; doi:10.1038/s41419-026-08726-w)
Supplement: Supplementary file 1 — Supplementary figure [file 41419_2026_8726_MOESM1_ESM.docx]

**
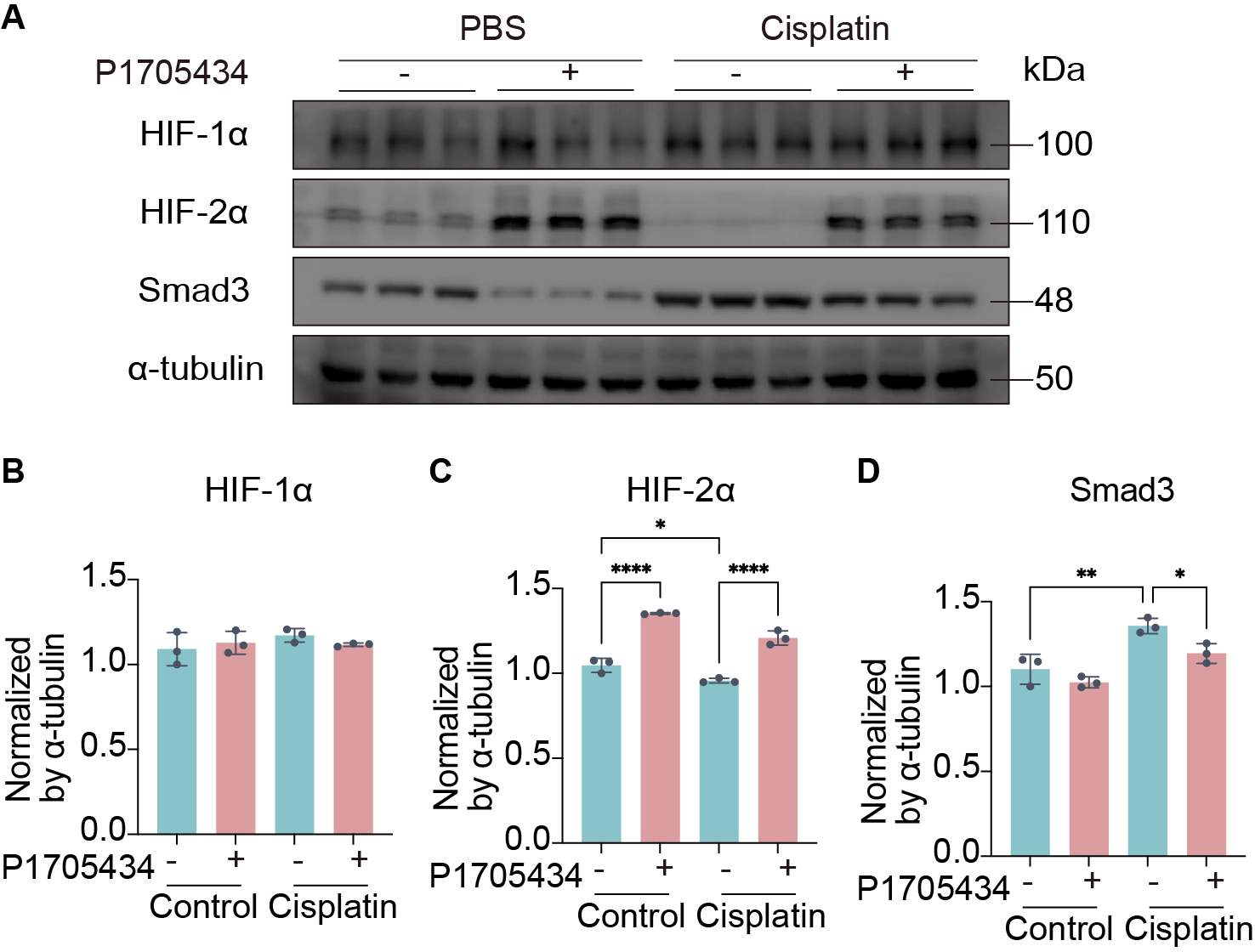
**

**Supplementary figure 1. Validation of P1705434 targeted protein expression in HK2 cell line.**

(A) Western blot analyses of P1705434 targeted protein expression and quantification of (B) HIF-1α, (C) HIF-2α and (D) Smad3 in cisplatin treated HK-2 cell (n = 3). Data are means ± SD, *P < 0.05, **P < 0.01, ***P < 0.001.


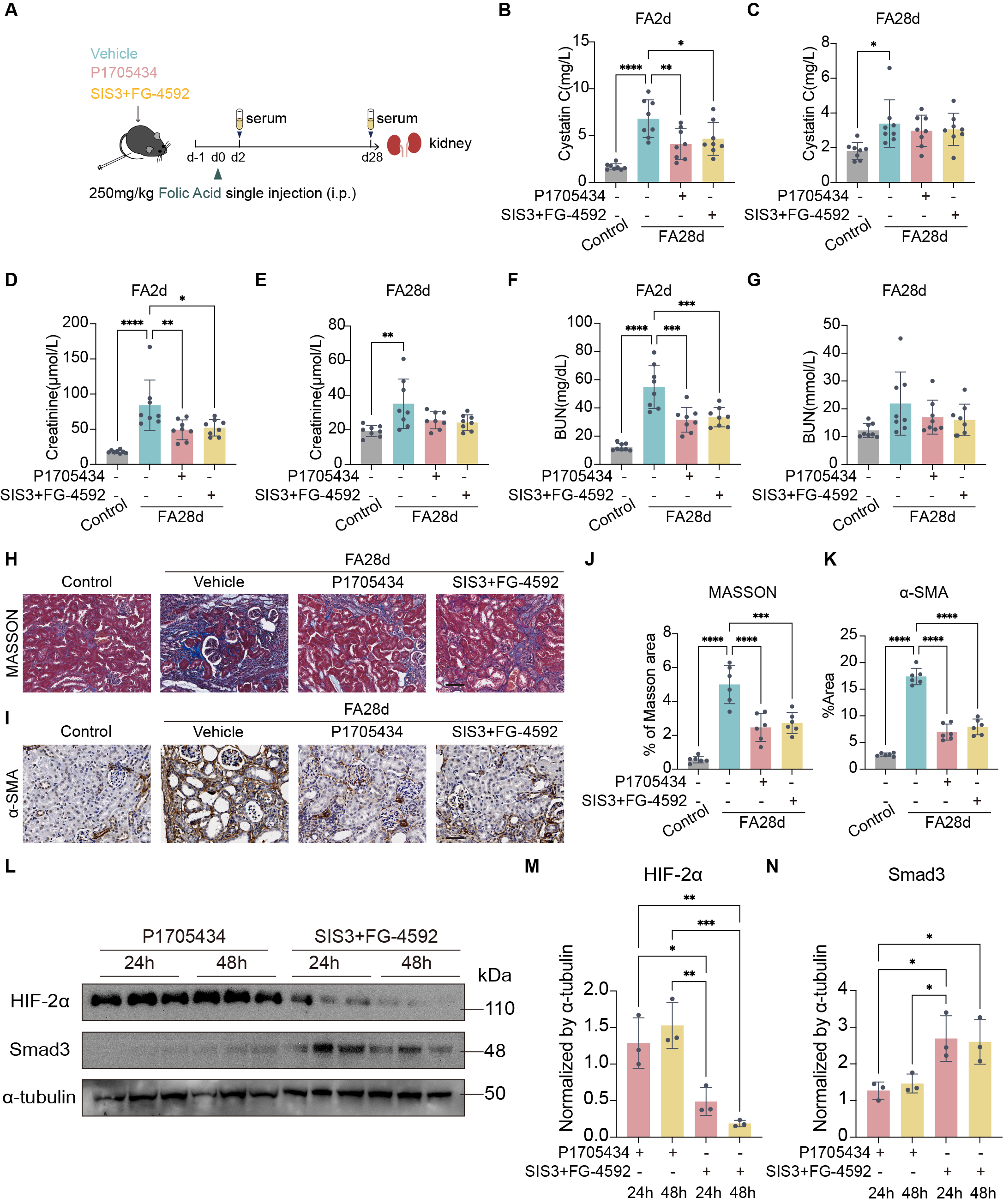


**Supplementary Figure 2. Comparable efficacy of P1705434 and SIS3+FG-4592 combination therapy in FAN mice.**

(A) Schematic of the experimental design for the comparative study in the FAN model. Renal function was evaluated with (B, C) Cystatin C, (D, E) serum creatinine and (F, G) BUN at day2 and day28 (n=8). (H) Representative images of Masson’s trichrome staining at day 28. (I) Representative IHC staining image of α-SMA among indicated groups at day 28. (J) Quantification of positive area of Masson’s trichrome and (K) α-SMA positive area at day 28. (n = 6). (L) Representative western blots showing protein levels of Smad3, HIF-2α, and α-tubulin at the indicated time points after drug removal. Quantification of (M) HIF-2α and (N) Smad3 protein levels were normalized to α-tubulin (n=3). FA = folic acid. Data are means ± SD, *P < 0.05, **P < 0.01, ***P < 0.001, ****P < 0.0001. Scale bars: 100 μm.


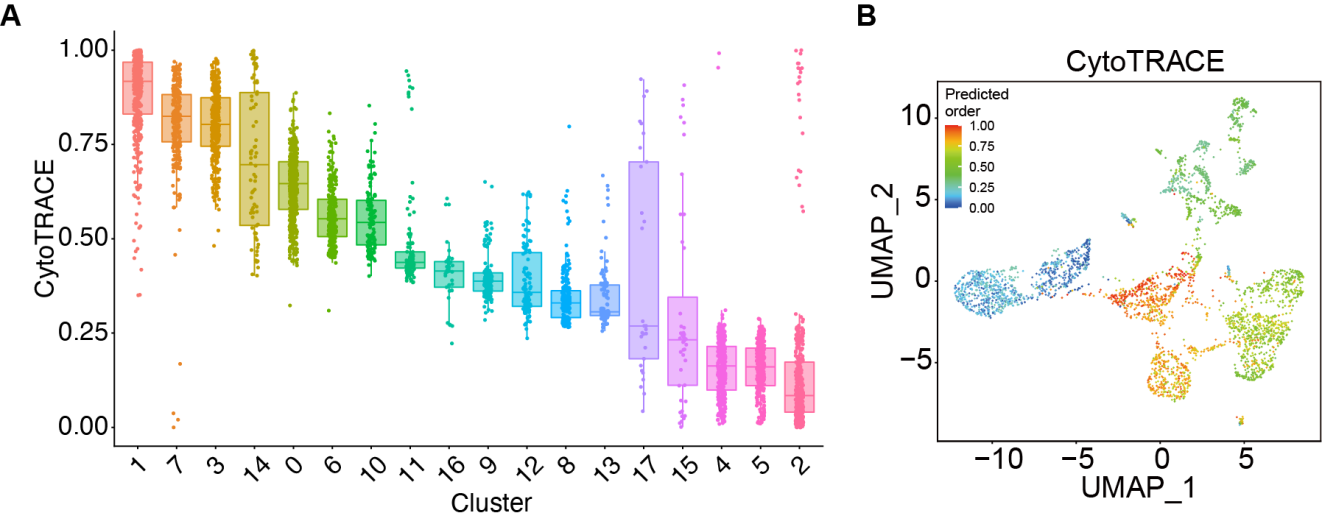


**Supplementary Figure 3. CD cells have the potential to differentiate into fibroblasts.**

(**A**) Stemness of all cells as determined by CytoTRACE analysis. (**B**) The UMAP of collecting duct cells as determined by CytoTRACE analysis.


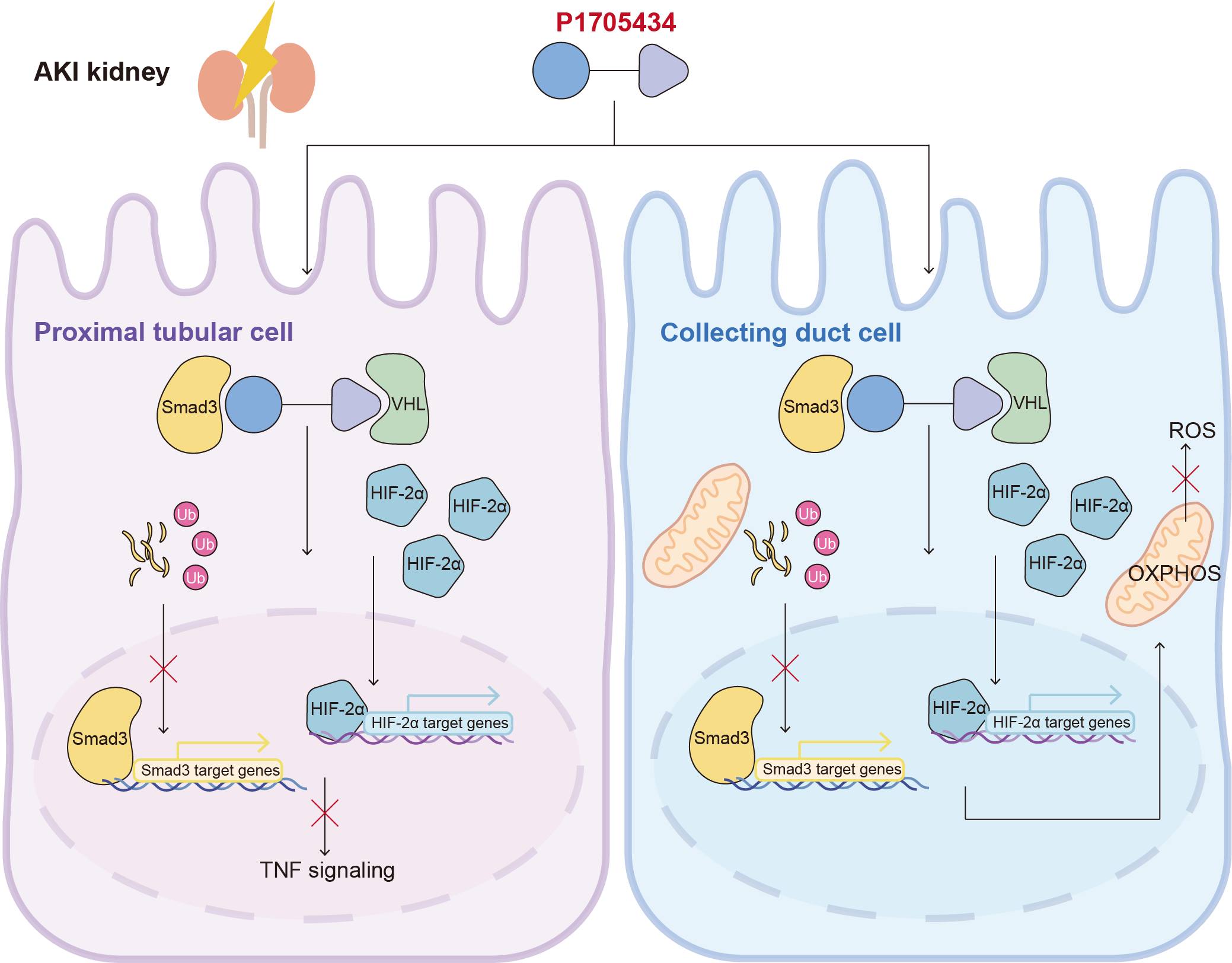


**Supplementary figure 4. Mechanism of VHL-recruiting PROTAC in AKI mice kidney.**

P1705434 down-regulates the TNF pathway to ameliorate cell injury in S3-proximal tubular cells, and it reduces the mitochondrial injury of collecting duct cells by up-regulating the OXPHOS pathway.
