# Supplementary material for: VHL-recruiting PROTAC attenuates AKI-CKD transition via simultaneous degradation of Smad3 and stabilization of HIF-2α
Source: Cell Death Dis. 2026 Apr 10;17(1):460. doi: 10.1038/s41419-026-08726-w (PMC13181044; doi:10.1038/s41419-026-08726-w)
Supplement: Supplementary file 2 — original western blots [file 41419_2026_8726_MOESM2_ESM.pdf]

Figure 2D

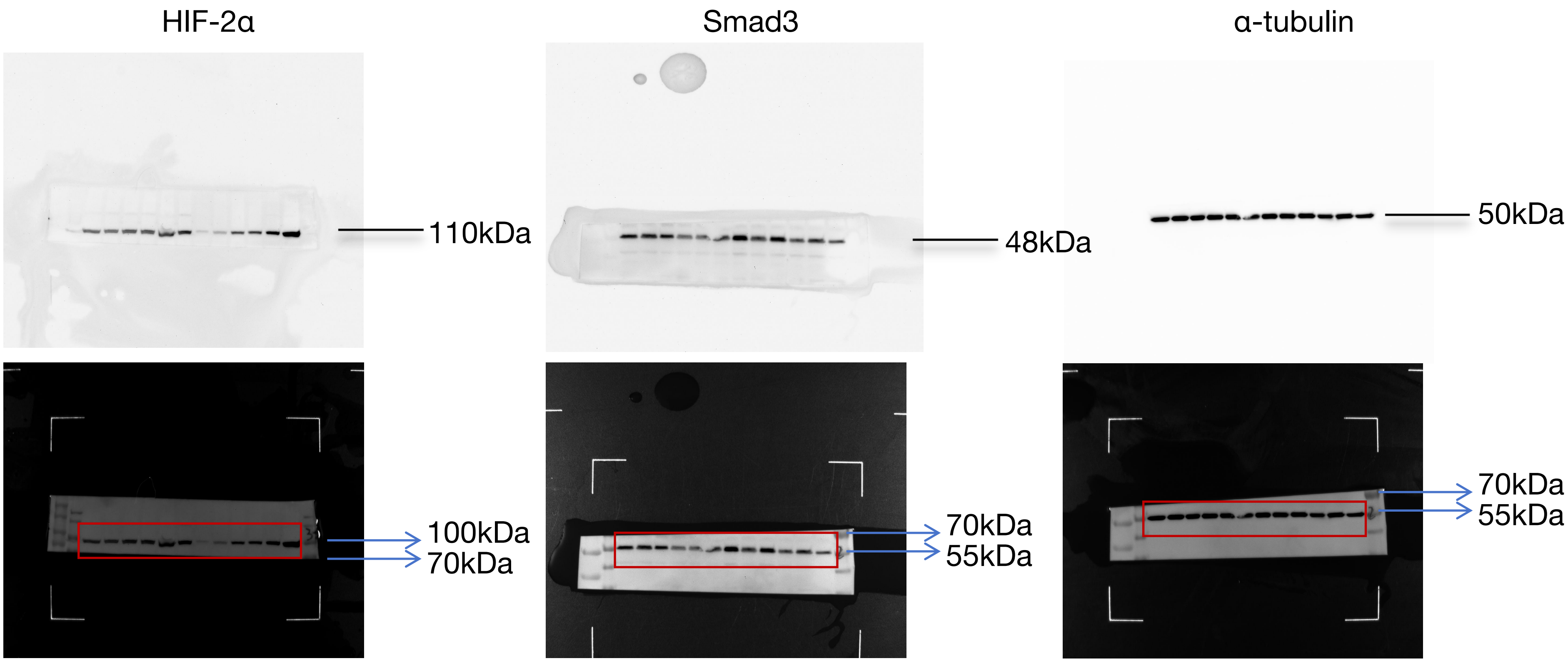

HIF-2α
Smad3
α-tubulin
110kDa
48kDa
50kDa
100kDa
70kDa
70kDa
55kDa
70kDa
55kDa

Figure 3G

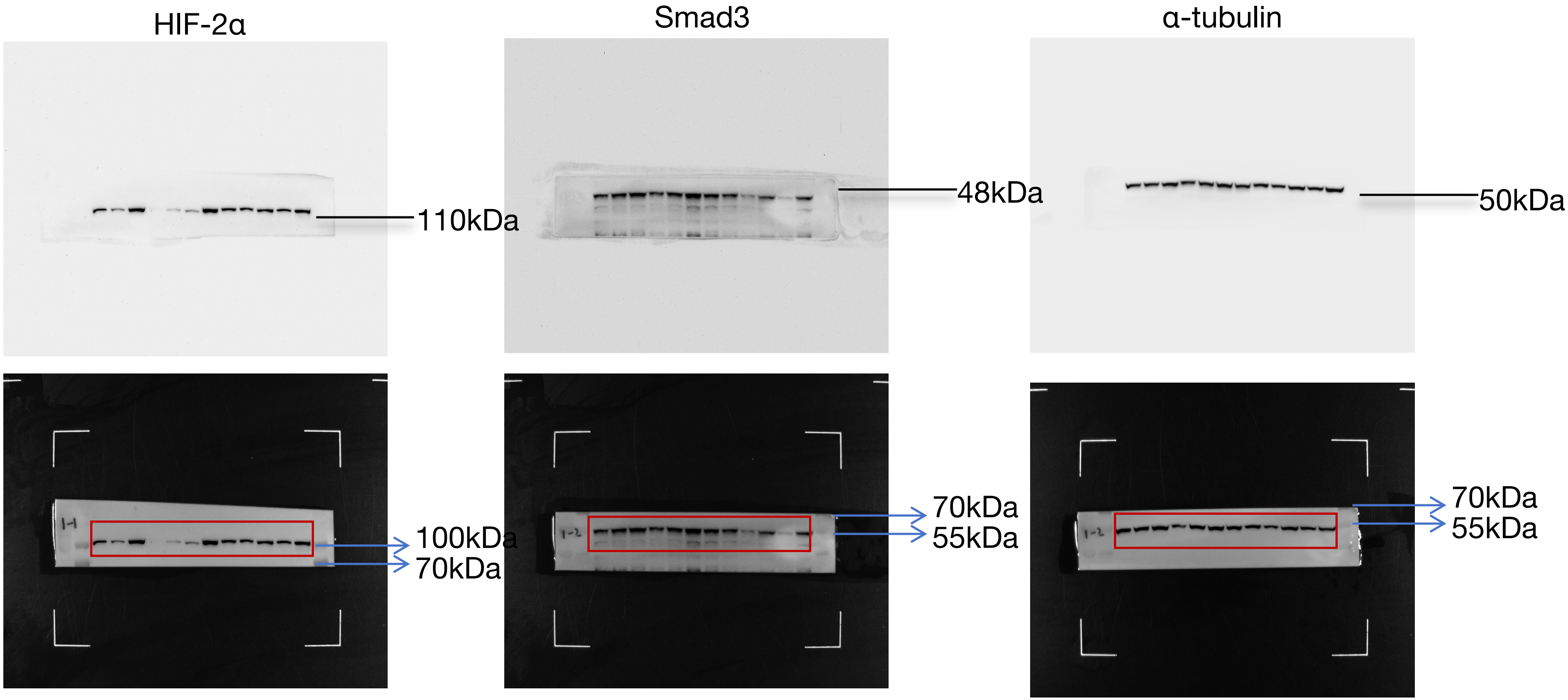
HIF-2α
Smad3
α-tubulin
110kDa
48kDa
50kDa
100kDa
70kDa
70kDa
55kDa
70kDa
55kDa

Figure S1A

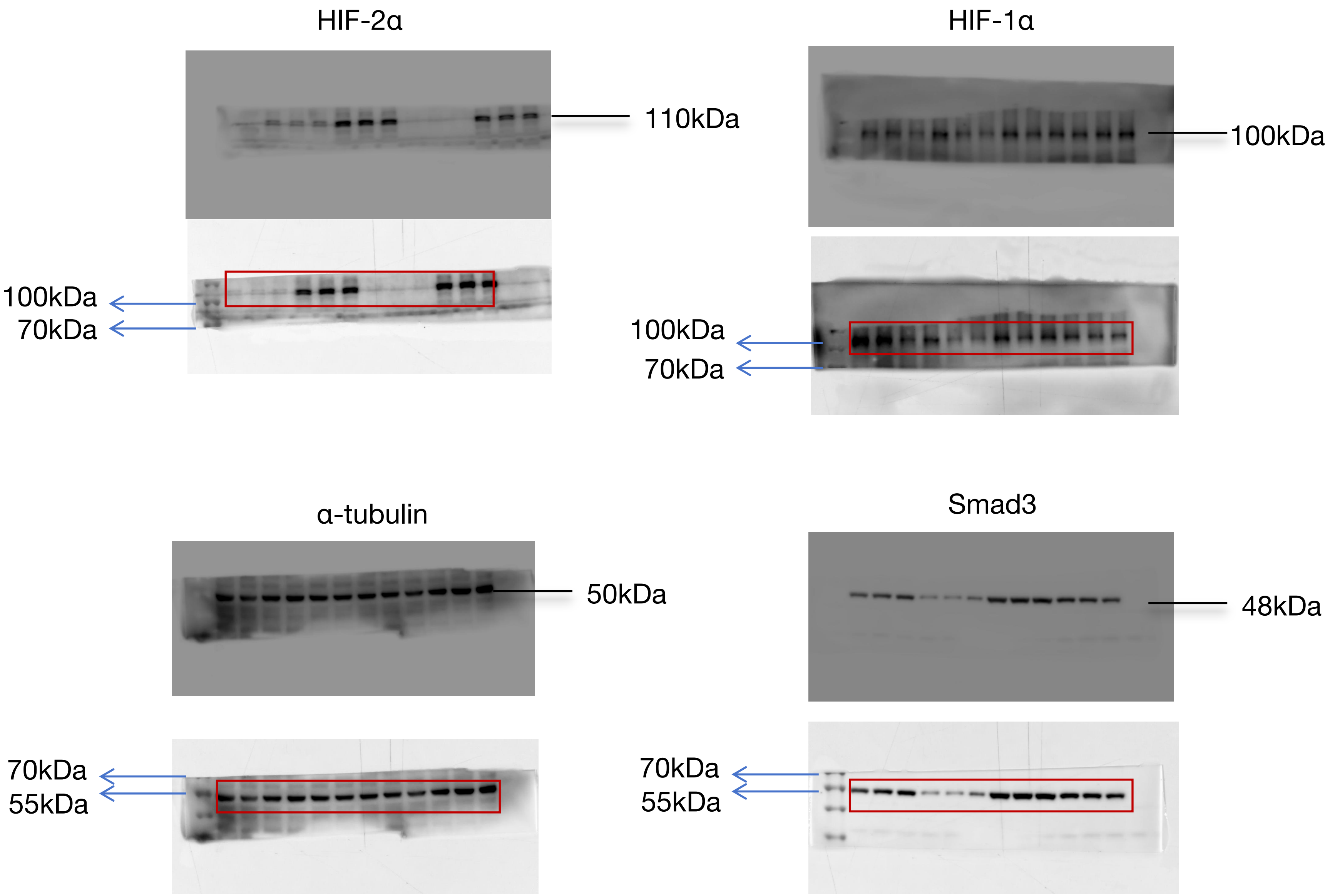
HIF-2α
110kDa
100kDa
70kDa
HIF-1α
100kDa
100kDa
70kDa
α-tubulin
50kDa
70kDa
55kDa
Smad3
48kDa
70kDa
55kDa

Figure S2L

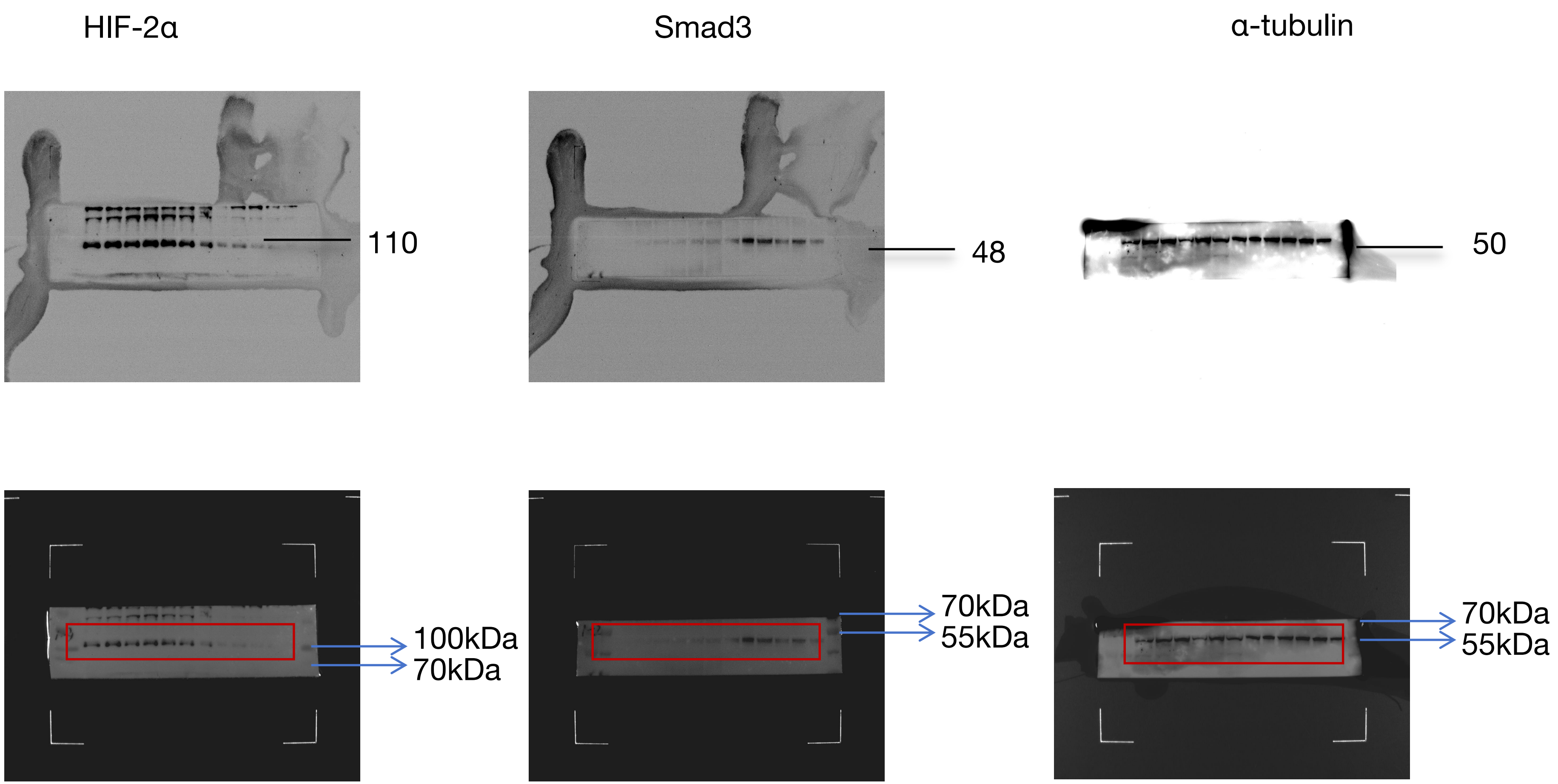
HIF-2α
Smad3
α-tubulin
110
48
50
100kDa
70kDa
70kDa
55kDa
70kDa
55kDa
